# Biofabrication of Composite Bioink‐Nanofiber Constructs: Effect of Rheological Properties of Bioinks on 3D (Bio)Printing and Cells Interaction with Aligned Touch Spun Nanofibers

**DOI:** 10.1002/adhm.202303343

**Published:** 2023-11-27

**Authors:** Waseem Kitana, Victoria Levario‐Diaz, Elisabetta Ada Cavalcanti‐Adam, Leonid Ionov

**Affiliations:** ^1^ Professorship of Biofabrication Faculty of Engineering Science University of Bayreuth Ludwig‐Thoma‐Straße 36A 95447 Bayreuth Germany; ^2^ Department of Cellular Biophysics Max Planck Institute for Medical Research Jahnstraße 29 69120 Heidelberg Germany; ^3^ Professorship of Cellular Biomechanics Faculty of Engineering Science University of Bayreuth Universitätsstraße 30 95447 Bayreuth Germany; ^4^ Bavarian Polymer Institute University of Bayreuth Universitätsstraße 30 95447 Bayreuth Germany

**Keywords:** 3D biofabrication, bioink, cell motility, cell orientation, extrusion 3D (bio)printing, rheology, touch‐spinning

## Abstract

This paper reports on a novel approach for the fabrication of composite multilayered bioink‐nanofibers construct. This work achieves this by using a hands‐free 3D (bio)printing integrated touch‐spinning approach. Additionally, this work investigates the interaction of fibroblasts in different bioinks with the highly aligned touch‐spun nanofibers. This work conducts a comprehensive characterization of the rheological properties of the inks, starting with low‐strain oscillatory rheology to analyze the viscoelastic behavior, when the material structure remains intact. Moreover, this work performs amplitude sweeps to investigate the stability of the inks under large deformations, rotational rheology to examine the shear thinning profile, and a three‐step creep experiment to study time‐dependent rheological behavior. The obtained rheological results are correlated to visual observation of the flow behavior of inks. These behaviors span from an ink with zero‐shear viscosity, very weak shear thinning, and no thixotropic behavior to inks exhibiting flow stress, pronounced shear thinning, and thixotropy. It is demonstrated that inks have an essential effect on cell behavior. While all bioinks allow a preferred directionality of the fibroblasts along the fiber direction, cells tend to form aggregates in bioinks with higher viscosity, and a considerable number of agglomerates are observed in the presence of laponite‐RD.

## Graphical abstract

Biofabrication of composite bioink‐nanofibers constructs resembles the complex architecture of tissue extracellular matrix. For this, 3D (bio)printing of inks integrated touch‐spinning of highly aligned nanofibers is investigated. The rheological properties of the inks and the cells interaction with the nanofibers show enhanced extrusion properties and cell motility and alignment along the fiber's main direction, respectively.

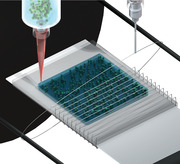

## 1 Introduction

Biofabrication, a multidisciplinary field that pursues the engineering of biologically relevant constructs, aims at replicating the complex architecture of human tissues and organs.^[^ ^1^ ^]^ Recently, biofabrication technologies have gained much attention on account of their possibilities of engineering tissues that would resolve the issue of donor organ and tissue deficit worldwide.^[^ ^2^ ^]^ A promising approach is the use of 3D (bio)printing, which is an advanced biofabrication technique that uses hydrogels to fabricate 3D biological constructs for tissue engineering applications.^[^ ^1^, ^3^ ^]^ Hydrogels have shown wide applicability in the fields of tissue engineering and biofabrication as scaffolding materials for decades. Furthermore, hydrogels are characterized by their high water retention ability, resembling the extracellular matrix (ECM) of various tissues.^[^ ^2^, ^4^, ^5^, ^6^, ^7^ ^]^ Thus, hydrogels provide the cells with an environment that fosters their proliferation and subsequent tissue maturation.

Various types of hydrogels have been extensively tested for 3D (bio)printing. Alginate‐based hydrogels are widely used in 3D (bio)printing as bioinks, in which different additives such as sodium carboxymethyl cellulose (SCMC) and Laponite‐RD have been used as rheological modifiers and shape retention enhancers to enhance alginate 3D printability.^[^ ^8^, ^9^, ^10^, ^11^ ^]^ Sodium carboxymethyl cellulose (SCMC) is a widely used water‐soluble biomaterial, which is usually used as a thickener in many industrial applications and as a bioink formulation either alone or blended with other materials such as alginate. Additionally, SCMC has also shown to be non‐toxic for cells, which show increased viability with time when blended with alginate.^[^ ^8^ ^]^ Laponite is water insoluble synthetic nano‐clay that forms a transparent colloidal gel in water through hydration and swelling of its particles, which is widely used in industry as a thickener. Moreover, laponite has been used in several tissue engineering applications showing great potential as a drug delivery carrier and hydrogel‐forming material, owing to its biodegradability and non‐toxic properties.^[^ ^9^, ^10^, ^11^ ^]^

However, hydrogels alone do not resemble or mimic the complexity of human tissue ECM, which is composed of fibrous proteins such as collagen and elastin and cell‐containing ground‐like (gel‐like) substances such as proteoglycans (PGs) and glycosaminoglycans (GAGs).^[^ ^2^, ^4^, ^6^, ^12^, ^13^, ^14^, ^15^, ^16^, ^17^ ^]^ Moreover, the softness and poor mechanical properties of hydrogels (brittleness) can hinder their applications for the fabrication of hard and/or high‐load tissues such as bone, myocardium, ligaments, cartilage, skeletal muscles, and many others.^[^ ^2^, ^5^, ^7^, ^12^, ^14^, ^17^, ^18^ ^]^ Additionally, hydrogels are bulk and isotropic in nature and do not mimic the anisotropic properties of many tissues that are natively provided by collagen fibrils in ECM, mostly resulting in non‐controlled cell orientation and arrangement. These anisotropic properties of directed fibers play a major role in cell attachment, proliferation, and maturation.^[^ ^12^, ^15^, ^16^, ^17^, ^19^, ^20^ ^]^ Additionally, anisotropy, which can be seen in the orientation of extracellular fibers can play a great role in the proper functioning of many native tissues such as myocardium, skeletal muscles, and corneal stroma.^[^ ^2^, ^5^, ^14^, ^15^, ^19^, ^21^ ^]^ For instance, the myocardium is composed of muscular fibers that are highly oriented, in which fiber orientation is essential for both the mechanical and electrical functioning of the heart.^[^ ^19^, ^22^ ^]^ Another example, is bone, which is composed of aligned collagen fibers along the bone length that play a great role in both its mechanical and biological properties.^[^ ^16^ ^]^ A further instance is the corneal stroma, which consists of perpendicularly aligned and tightly packed collagen fibers that play an essential role in the transparency of the stroma.^[^ ^2^, ^23^ ^]^ The fibrocartilaginous meniscus is an additional example, where the orientation of collagen fibers is crucial for its mechanical and biological properties. Natively, the meniscus is composed of peripherally oriented fiber bundles, which gives rise to the anisotropic properties as well as to the extremely high tensile strength of the meniscus in the main direction of fibers.^[^ ^7^, ^24^, ^25^ ^]^ Another tissue, where the alignment of fiber and hence cellular alignment is of great importance in tissue normal functioning is the skeletal muscles. Native skeletal muscles are composed of parallel bundles of collagen fibers also called myofibers where the myoblast cells (precursor muscle cells) are tightly packed and highly aligned along the collagen fiber's main direction forming what is known as myotubes. This alignment of collagen fibrils is necessary for the proper mechanosensing of myoblasts differentiation as well as subsequent synchronized contraction of the myofibers of the muscular tissue.^[^ ^26^, ^27^, ^28^, ^29^ ^]^ For that reason, it is of high importance to biofabricate constructs that match the native orientation or alignment of collagen fibers in native tissues, meanwhile maintaining cell viability and orientation within a hydrated 3D environment.^[^ ^15^, ^16^ ^]^

A wide range of approaches and techniques has been proposed to combine 3D (bio) printing of bioinks or biomaterial inks with nanofiber fabrication techniques such as electrospinning ^[^ ^3^, ^7^, ^13^, ^14^, ^16^, ^18^, ^30^, ^31^ ^]^ and melt electrowriting (MEW) ^[^ ^2^, ^4^, ^5^, ^32^ ^]^ for the fabrication of hydrogel‐fiber constructs.^[^ ^2^, ^4^ ^]^ However, the use of electrospinning and melt electrowriting have intrinsic drawbacks, which limit their applicability in combination with 3D (bio) printing, in which the cells are usually incorporated –, for example, 3D bioprinted – post‐fiber fabrication in a multi‐step manner.^[^ ^32^ ^]^ For instance, electrospinning, which is the most commonly used technique for the fabrication of nanofibers, usually produces fibrous mats with randomly aligned fibers on a stationary substrate with non‐controlled 3D structures.^[^ ^2^, ^4^, ^15^, ^16^, ^33^ ^]^ Moreover, usually the production of relatively aligned fibers requires the use of a rotating mandrel with high rotational speeds, which limits its use in combination with 3D (bio) printing.^[^ ^16^ ^]^ Another approach to produce aligned fibers is by using a two‐bar setup that allows the deposition of uniaxially aligned fibers but is, however, rather uncontrolled. Furthermore, the control over the mesh architecture such as the fiber directionality and spacing between fibers is limited.^[^ ^2^, ^5^, ^6^, ^33^, ^34^ ^]^ Additionally, electrospinning uses extremely high voltages in the range of a few kV that hinder its wide applicability in combination with 3D (bio) printing of bioinks, as it could have detrimental effects on cell viability and other bioactive molecules.^[^ ^21^, ^35^, ^36^ ^]^ Finally, electrospun mats are usually incorporated into already 3D (bio) printed hydrogel layers by manual intervention in a multistep process, which makes it difficult to handle especially when using thin fibrous mats.^[^ ^7^, ^12^ ^]^ On the other hand, melt electrowriting is able to produce well‐controlled 3D fibrous constructs in a programmed deposition manner that allows for its integration with 3D (bio) printing.^[^ ^4^, ^32^, ^33^, ^37^ ^]^ However, melt electrowriting is a relatively slow fabrication method with low output due to the high viscosity of the polymer melt used and charge dissipation with increased thicknesses.^[^ ^16^, ^33^, ^37^, ^38^ ^]^ A combined disadvantage of electrospinning and melt electrowriting is the difficulty of deposition of fibers on thick non‐conductive substrates. Thereby, touch‐spinning was used, which is based on mechanically drawing nanofibers from polymer solutions and/or melts to produce highly aligned fibers on a stationary substrate with fiber diameter in the range from a few nanometers to a few micrometers.^[^ ^21^, ^35^, ^36^ ^]^ This simple and scalable fabrication method is based on highly rotating fiber drawing bars that mechanically pull the fibers from a polymer solution and/or melt droplet formation on the tip of the needle of the syringe and deposit the fibers on a stationary or rotating substrate to yield fibers in different orientations. Solution touch‐spinning is mainly based on complete solvent evaporation due to the high volatility of the solvent and the increase in the surface area of the droplet while rotating and extending the fiber. On the other hand, melt touch‐spinning is based purely on the mechanical stretching of the melt droplet and subsequent cooling of the melt while rotating.^[^ ^20^, ^21^, ^34^, ^35^, ^36^ ^]^ Moreover, touch spinning is a relatively fast fabrication method with a relatively high output compared to electrospinning and melt electrowriting.^[^ ^33^, ^35^, ^36^ ^]^ From the technical point of view, this will allow for the hands‐free combination of the fiber deposition method with 3D (bio) printing, where nanofibers and bioinks can be deposited in a sequential alternating manner to fabricate a multilayered construct. The use of a combination of a hydrogel‐based system with a fiber system, reduces the requirements for the processing of hydrogels such as crosslinking to increase their mechanical properties, since in these composites the mechanical properties are covered by the fiber system. Additionally, the demand for a low crosslinking degree is advantageous for cell migration and subsequent tissue formation.^[^ ^5^, ^7^, ^31^ ^]^

Herein, in this study, a novel biofabrication technique, which is based on integrating 3D (bio) printing and touch spinning to fabricate a multilayered construct of cells encapsulated hydrogel system with highly aligned nanofibers was investigated. We have used biomaterial inks that are widely used for the fabrication of composite constructs: alginate, carboxymethyl cellulose, and laponite as the main component of bioinks and polycaprolactone as fiber spinning polymer. The hydrogel will provide the cells with an aqueous environment that fosters cells well‐functioning whereas fibers are expected to guide cell alignment along the fiber's main direction. Alginate solutions are fluidic in nature – they flow at relatively low stress, where the flow rate is dependent on the concentration of the polymer solution and the applied stress, which in most cases is gravity. This kind of flow results in the spreading of the 3D‐printed samples. We used different additives, which influence the rheological properties of alginate solutions to make them more stable following 3D (bio) printing. The rheological behavior of biomaterial inks is, however, important not only from the point of view of providing structural stability of 3D printed structures, it also affects the motility of cells in the bioink. This is essential for the cellular interactions with the nanofibers and the ability of the cells to sense or reach the fibers and align along their main direction. Hence, in this paper, we report on the fabrication of composite bioink‐nanofiber constructs and correlated behavior of cells, their alignment along the fibers as well as 3D printing in general with the rheological properties of the different alginate‐based biomaterial inks (alginate alone, alginate with SCMC and/or laponite).

## 2 Results and Discussion

**Figure 1:**
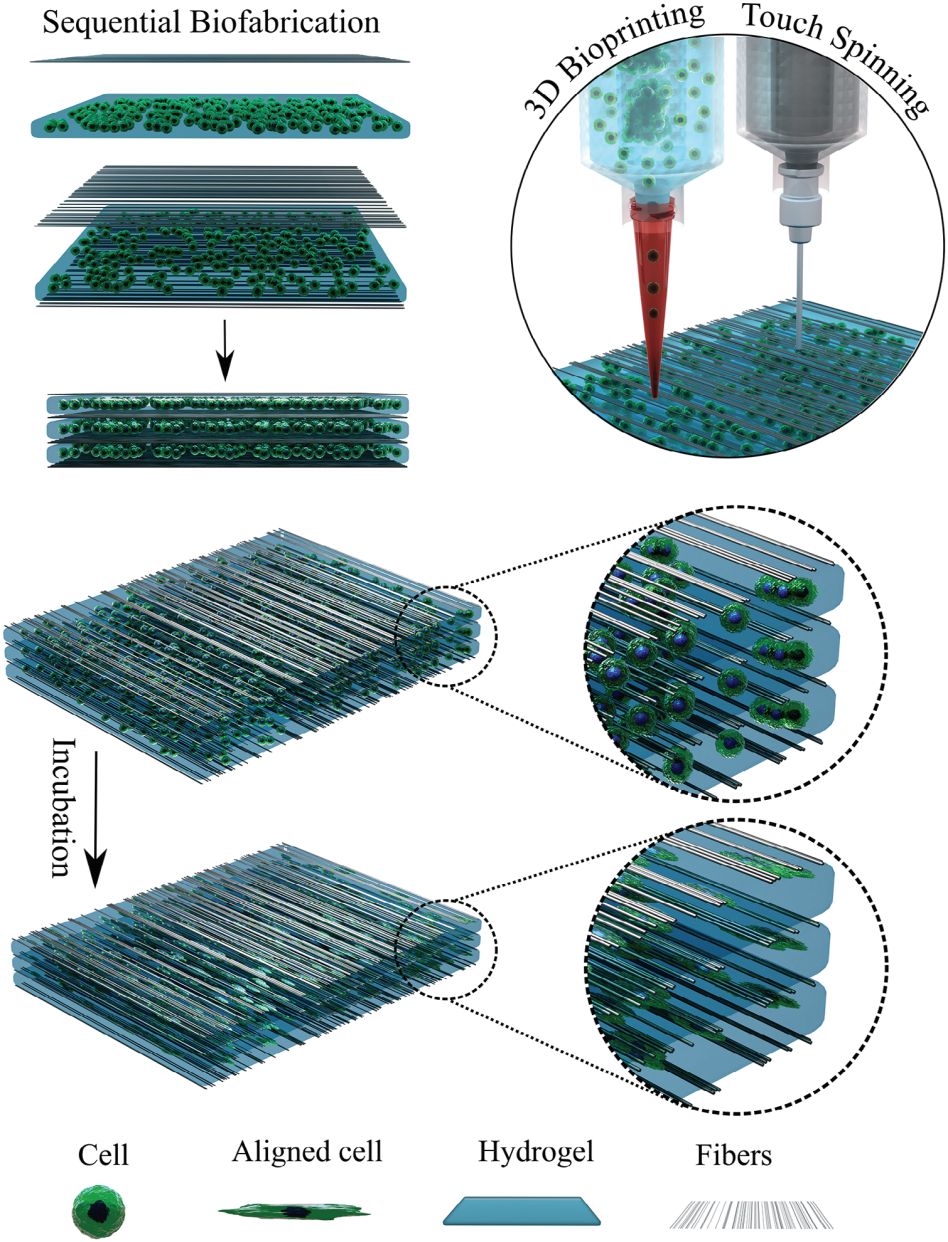
Schematic representation of the fabrication process of hydrogel‐cell (bioink) – nanofibers construct using the hands‐free 3D (bio) printing of bioinks integrated touch‐spinning of nanofibers.

In this paper, we studied the effect of the rheological properties of the biomaterial inks on interactions of fibroblasts with fabricated constructs, which were chosen as a model system, with nanofibers embedded in bioink‐formed hydrogel. Fibroblasts are a type of cells that are found throughout the human body in connective tissues and many other tissues such as the dermis and the myocardium. This type of cells are responsible for the secretion of fibrous proteins such as collagen and elastin and in turn, synthesize the ECM of many tissues and play a major role in wound healing in case of injury by migrating to the damaged tissue.^[^ ^39^, ^40^ ^]^ The hydrogel‐fiber composites were prepared as follows; first aligned polycaprolactone (PCL) nanofibers were fabricated using touch spinning followed by 3D (bio) printing of the bioink and this was repeated sequentially (hands‐free) in a layer‐by‐layer manner to fabricate a multilayered nanofiber – bioink composite construct (**Figure** **1**).

Several biomaterial inks (without cells) were considered, and their rheological properties were first studied thoroughly. Thus, three hydrogel‐forming biomaterial inks with different rheological properties: alginate, sodium carboxymethyl cellulose (SCMC), and laponite were selected. Additionally, blends of these polymer solutions (alginate with SCMC, alginate with laponite, and alginate with SCMC and laponite) were also studied. Moreover, pure water and cell culture media were used to study the effect of the ionic strength of the solvent on the rheological behavior of the polymer solutions. In total, in this study, six hydrogel‐forming polymer solutions were dissolved in two different solvents, pure water and cell culture media.

**Figure 2:**
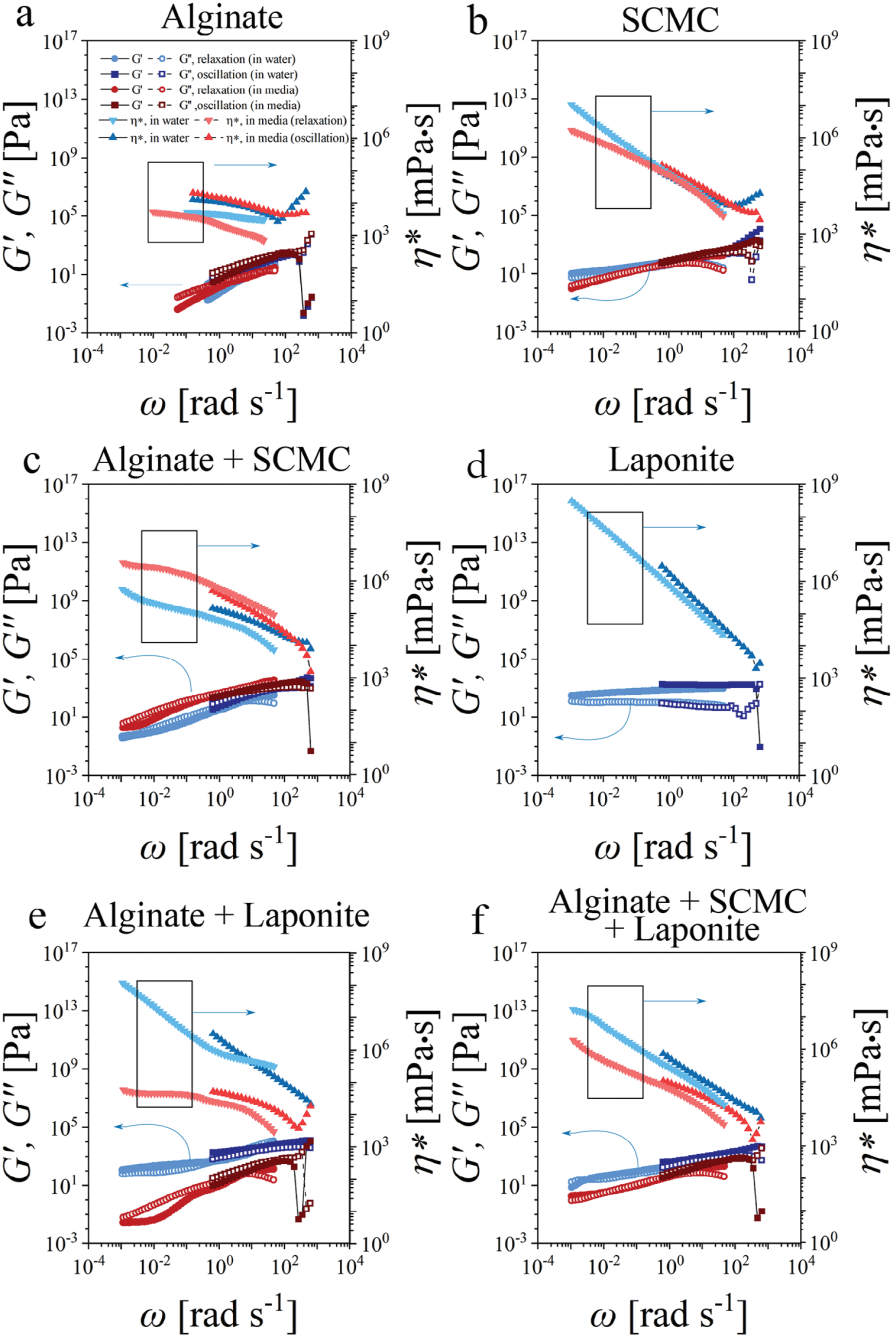
Viscoelastic behavior of the biomaterial inks (storage modulus (*G*′) and loss modulus (*G*′′) versus angular frequency (ω)) obtained from oscillation (shear strain, γ = 0.1%) and relaxation experiments (shear strain, γ = 10%) in water and cell culture media for a) pristine alginate 3.5% (w/v), b) pristine sodium carboxymethyl cellulose (SCMC) 3% (w/v), c) blended alginate 3.5% (w/v) with SCMC 3% (w/v), d) pristine laponite‐RD 5% (w/v), e) blended alginate 3.5% (w/v) with laponite 5% (w/v), and f) blended alginate 3% with SCMC 1.5% (w/v) and laponite‐RD 1.5% (w/v) (ratio 2:1:1 (w/w)).

**Figure 3:**
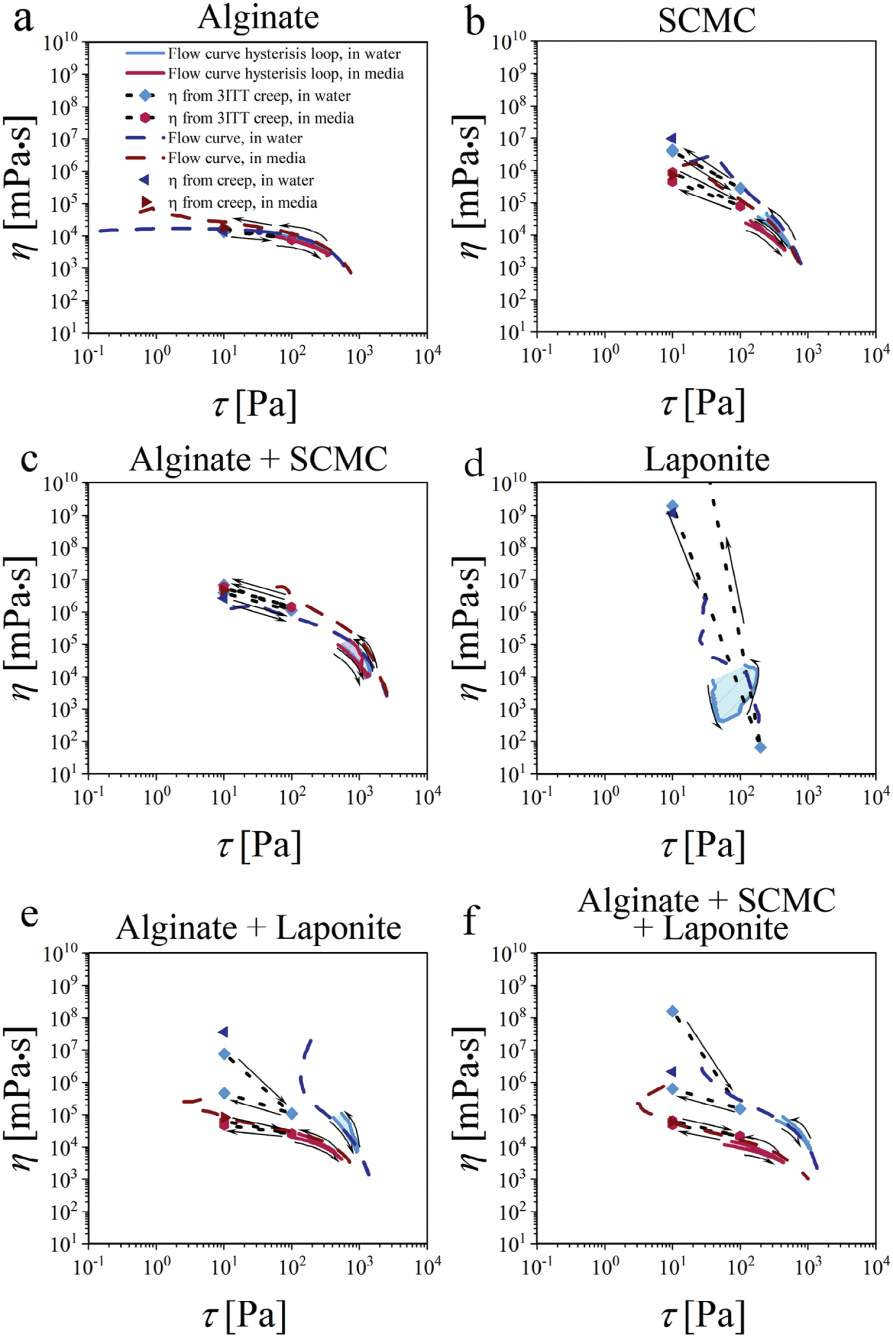
Hysteresis loop (shear rate ramp up‐constant‐down) and steady‐state shear rate ramp (viscosity (η) versus shear stress (τ)) with apparent viscosities from creep‐recovery and three interval creep test (3ITT) in water and cell culture media for a) pristine alginate 3.5% (w/v), b) pristine sodium carboxymethyl cellulose (SCMC) 3% (w/v), c) blended alginate 3.5% (w/v) with SCMC 3% (w/v), d) pristine laponite‐RD 5% (w/v), e) blended alginate 3.5% (w/v) with laponite 5% (w/v), and f) blended alginate 3% with SCMC 1.5% (w/v) and laponite‐RD 1.5% (w/v) (ratio 2:1:1 (w/w)).

First, we studied the rheological properties of the biomaterial inks used, using oscillation and rotational rheology. For this, we started with studying the viscoelastic properties of the biomaterial inks at a small deformation (*γ* = 0.1%), when the intermolecular and or interparticle bonds are not broken during the measurement – structure and properties of the biomaterial remain intact. Relaxation experiments were conducted by applying larger deformation (*γ* = 10%) to achieve measurable forces. Moduli versus angular frequency plots obtained from oscillation (Figure S1, Supporting Information) and stress‐relaxation experiments (Figures S2–S10, Supporting Information) were combined (**Figure** **2**) to illustrate the relaxation process at a broad time scale, ranging from 10^−3^ to 10^3^ rad⋅s^−1^. First of all, alginate in water and cell culture media (Figure 2a) shows a linear drop in both moduli (storage (*G*′) and loss (*G′′*) modulus) with decreasing frequency in double log coordinates on a large time scale (*t* > 10^1^ s).^[^ ^41^ ^]^ Hence, this indicates a transition into a terminal flow regime, in which alginate is a viscous liquid with distinct zero‐shear viscosity.^[^ ^41^ ^]^ The obtained value of complex viscosity is nearly constant at a large time scale at 10^4^ mPa⋅s, which shall correlate to zero‐shear viscosity obtained from rotational experiments (**Figure** **3**, Figures S20 and S21, Supporting Information).

The behavior of SCMC in pure water and cell culture media is nearly identical – both moduli *G′* and *G′′* have nearly the same behavior with slight domination of *G′′* and slight decay of both curves with time (Figure 2b).^[^ ^42^ ^]^ Moreover, both moduli obtained for cell culture media are slightly reduced compared to that of SCMC dissolved in water due to the ionic strength of cell culture media that screens electrostatic charges of SCMC (Figure 2b). In contrast to alginate solutions, SCMC does not show a transition into a terminal flow regime on the time scale studied.^[^ ^42^ ^]^ Hence, this indicates the presence of a certain relaxation process in its structure over the studied time scale.

Laponite dispersion in water shows no stress relaxation behavior over the whole studied time range, in which both moduli are nearly independent of frequency with *G′* higher than *G′′* over the entire range of frequencies used.^[^ ^43^ ^]^ This indicates the pure elastic (gel‐like) behavior of laponite, although with considerable energy dissipation (*tan δ* = 0.1). On the other hand, in cell culture media, laponite tends to precipitate and does not form a colloidal dispersion, that is due to the high ionic strength of cell culture media that screens the electrostatic charges and hence particles agglomerate (Figure 2d). It is also well‐established that laponite is sensitive to the ionic properties of the solvent used.^[^ ^43^, ^44^ ^]^

The viscoelastic behavior of the blends, which include alginate + SCMC + laponite, alginate + SCMC, and alginate + laponite, can be understood as a combination of the behaviors exhibited by their individual components (Figure 2c,e,f). In water, the behavior at the short‐time scale is a combination of the behavior of their individual components and on the long‐time scale, the material behaves as a single component, which does not flow – either laponite or SCMC. In cell culture media, alginate + SCMC and alginate + laponite blends tend to undergo a transition to flow state – the constant value of complex viscosity at a low frequency that is due to screening of electrostatic interactions and breaking contacts between particles. Interestingly, blends with laponite (Figure 2e,f) are particularly sensitive to the ionic strength of the solvent.^[^ ^43^, ^44^ ^]^ They show significantly lower values of both moduli when dissolved in cell culture media compared to in water. Particularly, alginate blended with laponite is more sensitive to the ionic strength of the solvent, since as previously mentioned alginate flows at a large time scale and laponite does not form a colloidal gel in cell culture media.

Next, amplitude sweep measurements have been conducted to study the behavior of the biomaterial inks at large deformations and to identify the yield and flow points of the different hydrogel formulations. The yield point is the point, above which the material starts to change its viscoelastic properties, whereas above the flow point, the material starts to flow, that is, the transition from predominant elastic to the viscous state (Figures S11 and S12, Supporting Information).^[^ ^45^, ^46^ ^]^ All materials demonstrate a decrease in *G′* and *G′′* at large shear strains (Figure S11, Supporting Information) and shear stresses (Figure S12, Supporting Information), although the mode of the decrease is different. In terms of alginate, it shows the typical behavior of a polymer solution with *G′′* > *G′* and both moduli (*G′* and *G′′*) decrease gradually at large stresses (Figures S11 and S12a, Supporting Information). In the case of SCMC (Figures S11 and S12b, Supporting Information) and alginate blended with SCMC and laponite (Figures S11 and S12f, Supporting Information), they show the behavior of viscoelastic solid (*G′* > *G′′*) at small shear stresses and viscoelastic liquid (*G′* < *G′′*) at large shear stresses/strains.^[^ ^42^ ^]^ Similarly, alginate blended with SCMC shows nearly the same behavior of the viscoelastic solid (*G′* > *G′′*), even though the stress starts to decrease at large strains, which is an indication of material failure (Figures S11 and S12c, Supporting Information). On the contrary, pristine laponite in water shows the typical behavior of colloidal gel, where *G′* is much larger than *G′′* (*G′* >> *G′′*) at small deformations with a rapid increase in *G′′* near the yield point followed by a rapid and sharp decrease in loss modulus around the flow point (Figures S11 and S12d, Supporting Information). The behavior of alginate blended with laponite is the most complex among the others – its solution in pure water behaves as a combination of a colloidal gel and a polymer solution (Figures S11 and S12e, Supporting Information). This is indicated by that, *G′′* > *G′* at small shear stress/strain with a rapid drop of *G′* at larger shear stresses, due to failure of particle network and finally a gradual decrease of both moduli (*G′′* > *G′*) with increasing shear stress or strain. On the other hand, alginate blended with laponite in cell culture media behaves in a similar way to pristine alginate. This can be explained by the screening of electrostatic interactions between laponite particles and their inability to form a network.

Thus, all formulations in water show the typical behavior of polymers with a steady state or constant change in both moduli up until the yield point (linear viscoelastic (LVE) region) followed by a gradual decrease in both moduli above the yield point. One exception is laponite, which shows a rapid increase in loss modulus (*G′′*) near the yield point followed by a rapid and sharp decrease in loss modulus around the flow point (weak strain overshoot) forming a distinct peak (Figures S11 and S12d, Supporting Information). This indicates a sudden change in the flow properties of laponite. Furthermore, this points out that there is microcracks formation shortly before the yield point accompanied by a resistance against flow followed by macrocracks development in the overall structure of laponite above the yield point, where the material starts to flow.^[^ ^47^ ^]^ Generally, all formulations show a rapid fall in storage modulus above the flow point compared to loss modulus which is due to breakage of intermolecular bonds that initiate the flow of the material.

Amplitude sweep was also used to study the effect of the ionic strength of the solvent on the viscoelastic properties of the hydrogels. First of all, cell culture media shows a minor effect on the viscoelastic behavior of alginate, SCMC, and alginate mixed with SCMC (Figures S11 and S12a–c, Supporting Information). Whereas, in the case of alginate with laponite and alginate mixed with SCMC and laponite, it shows in the LVE region a detrimental change in the viscoelastic properties from elastic‐like behavior in water into a viscous‐like behavior in media with a decrease in both moduli (Figures S11 and S12e,f, Supporting Information). Thus, this can be explained by that laponite does not form a network in cell culture media, and hence its contribution to rheological properties is reduced. Hence, it can be concluded that laponite‐based bioinks are strongly influenced by the ionic strength of the solvent.

Thus far, it can be concluded that the high ionic strength of cell culture media has a minor effect on the viscoelastic behavior of pristine alginate, pristine SCMC, and alginate mixed with SCMC. In contrast, the rheological behavior of laponite‐based blends (alginate + laponite and alginate + SCMC + laponite) is strongly affected by the ionic strength of the solvent, in which the rheological properties of laponite‐based blends are reduced in cell culture media.

Next cyclic thixotropy was conducted. Cyclic thixotropy is one of the fundamental aspects of studying biomaterial ink printability. It indicates the transition kinetics of the material properties from solid‐like to fluid‐like behavior and back, over several cycles of extrusion for the proper elastic shape retention of the 3D (bio) printed biomaterial ink. This also gives information about the stability of the biomaterial ink over time under cyclic shearing. For this, the thixotropic behavior was studied using two approaches: investigation of (i) time‐dependent viscoelastic properties of hydrogel formulations under cyclic changes in applied shear strain/stress (Figures S13–S16, and Tables S1–S4, Supporting Information) and (ii) dependence of shear viscosity on shear rate/stress under shear rate ramp up‐constant‐down (Thixotropic loop) (**Figure** **3**, Figures S22 and S23, Supporting Information). The fundamental difference between the two methods is that the first one addresses the viscoelastic behavior of the biomaterial inks within a very narrow time scale, whereas the second one shows how the flow rate changes when applied stress increases and then decreases back.

Figure S14, Supporting Information shows changes in the loss and storage modulus under cyclic changes in shear strain within the linear viscoelastic (LVE) region (*γ* = 0.1%) and way beyond the yield point (*γ* = 1000%). Pristine alginate, pristine SCMC, and alginate + SCMC show immediate recovery of both moduli (*G′* and *G′′*) to their initial values following large deformation (*γ* = 1000%) independent from the solvent used (Figures S13 and S14a,b,e, Supporting Information). Similarly, pristine laponite in water shows an instant recovery of both moduli after large deformations applied over several cycles (Figures S13 and S14d, Supporting Information). As previously stated, laponite was not able to form a colloidal gel in cell culture media and thus its rheological properties in cell culture media were not studied. Furthermore, the recovery properties of laponite‐based blends (alginate + laponite and alginate + SCMC + laponite) are the most complicated (Figures S13 and S14e,f, Supporting Information). The recovery of these blends in cell culture media is nearly immediate and complete. Whereas the recovery of these blends in water is not thorough and relatively slow. Thus, the recovery kinetics can be visually observed and the values of *G′* and *G′′* achieved decreases with increasing number of cycles. This effect is particularly visible for alginate + laponite in water (Figure S14e, Supporting Information). Hence and as previously discussed, this recovery behavior of laponite‐based blends is essentially due to that laponite determines the rheological properties of its blends in water. As stated, laponite in a low ionic strength environment (in water) forms a gel whereas in a high ionic strength environment (in cell culture media), electrostatic interactions between its particles are screened. Hence the rheological properties of the laponite blends in media are governed by other components of the blend such as alginate and alginate + SCMC in this case. In low ionic strength solvents, all the individual components (alginate, SCMC, and laponite) determine the overall recovery process and kinetics. Further, alginate as a polymer solution is, however, a viscous substance that retards the movement of laponite particles and hence agglomerates and retards the recovery process. It can be concluded that alginate, SCMC, and alginate + SCMC in water and media as well as laponite in water show structure recovery under cyclic changes in shear strain. Alginate + SCMC + laponite in water and media as well as alginate + laponite in media show a slight thixotropic behavior – weak history‐dependent rheological properties. In addition to that, alginate + laponite in water show structure loss over the cycles – strong thixotropy, which is explained by that alginate as a polymer hinders or retards the recovery of laponite as a colloidal gel.

We have also used rotational rheological measurements to assess the change in flow rate upon cyclic changes in stress (Figure 3 and Figures S19–S23, Supporting Information). For this, the shear rate was gradually ramped up and down to study the low‐shear‐rate viscosity recovery after high deformation of the material structure (Figure 3, Figures S22 and S23, Supporting Information). Additionally, viscosity was calculated from three interval creep (3ITT; 10 Pa, 100 Pa, 10 Pa) (Figure S18, Supporting Information) as well as two interval creep‐recovery tests (Figure S17, Supporting Information) using Equation S2, Supporting Information. These experiments were conducted to study the shear thinning behavior due to the breakage of the material structure and to study the reversibility of these structural changes.

Alginate in water and media show distinct zero‐shear viscosity (≈17 Pa⋅s) behavior with relatively weak shear thinning behavior at high shear stress (>30 Pa)/shear rate (>20 s^−1^) corresponding to the reciprocal relaxation time of polymer chains as well as satisfactory structure recovery, in which the viscosity had a minor change in its value after ramping back to the initial deformation (Figure 3, Figures S20 and S23a, Supporting Information). SCMC and alginate + SCMC in water and media show more pronounced shear thinning behavior with no distinct zero‐shear viscosity (≈10^6^ – 10^7^ Pa⋅s at rate of 0.01 s^−1^) (Figure 3, Figures S20 and S23b,c, Supporting Information). Furthermore, the recovery of the viscosity after high shear rate deformation is considerable. On the other hand, laponite in water shows very strong shear thinning behavior (viscosity drops down by 7–8 orders of magnitude) due to the break of contacts between the particles (Figure 3 and Figure S20d, Supporting Information). In addition to that, there is no hysteresis observed due to fast recovery of the structure after high‐stress application. Laponite‐based blends (alginate + laponite and alginate + SCMC + laponite) in water show also strong shear thinning behavior but incomplete viscosity recovery after high‐stress application (>50 Pa) – well‐pronounced thixotropic behavior. Similar to alginate, laponite‐based blends in cell culture media show less pronounced shear thinning behavior with nearly complete recovery of their viscosity after high‐stress application. This thixotropic behavior of aqueous solutions of laponite‐based blends is due to the break of contacts between particles and their relatively slow recovery is due to the high viscosity of laponite (Figure 3, Figures S20 and S23e,f, Supporting Information). In cell culture media, the contacts between laponite particles are weak. This results in lower viscosity and the absence of thixotropy – contacts between particles do not recover. In summary, performed rheological test elucidated the fundamental difference in mechanical and flow properties of the biomaterial inks. Alginate is a typical viscous polymer, while SCMC has combined properties of viscous polymer and colloidal gel with prominent shear thinning behavior. On one hand, alginate blended with SCMC is nearly identical to pristine SCMC although SCMC contributes with high viscosity. On the other hand, laponite may introduce shear thinning as well as thixotropic behavior depending on the solvent used and what kind of polymer is blended with it.

It is important to note that the stresses used in creep experiments 10 and 100 Pa correspond to gravity force acting on the lower side of the sample with 1 and 10 mm thick material layers, respectively, with a density of 1 g⋅cm^−3^ – typical density of polymer solutions and gels. In addition to that, the values of viscosity allow the estimation of the rate of biomaterial ink spreading. For instance, the shear rate of a 1 mm‐large droplet with a viscosity of 10^4^ mPa⋅s (pristine alginate) and 10^7^ mPa⋅s (pristine SCMC) attached to a vertical wall without considering surface tension of surfaces are 1 and 10^−3^ s^−1^, respectively. These values of shear rate correspond to a shear strain of 3600 (360 000%) and 3.6 (360%) obtained within 1 h of flow. Thus, this means that these values of viscosities are too low to ensure the stability of the 3D (bio) printed biomaterial ink without crosslinking or any other stabilization methods. Only pure laponite in water with viscosity >10^9^ mPa⋅s is able to form a stable structure – shear strain after 1 h is less than 3.6%.

**Figure 4:**
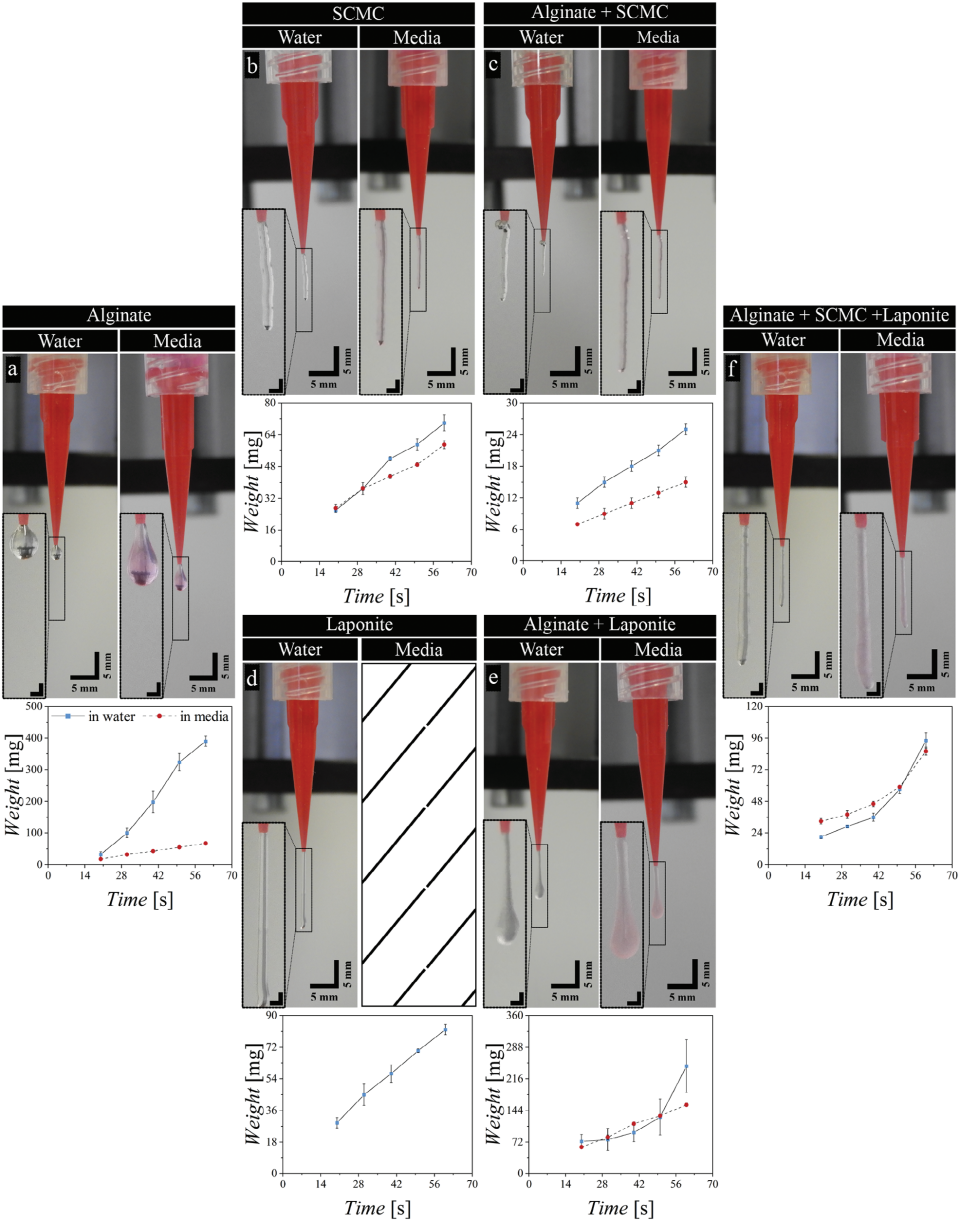
Suspended extrusion test and mass flow, (weight (wt) versus time (t)) of the extruded biomaterial inks in water and cell culture media for a) pristine alginate 3.5% (w/v), b) pristine sodium carboxymethyl cellulose (SCMC) 3% (w/v), c) blended alginate 3.5% (w/v) with SCMC 3% (w/v), d) pristine laponite‐RD 5% (w/v), e) blended alginate 3.5% (w/v) with laponite 5% (w/v), and f) blended alginate 3% (w/v) with SCMC 1.5% (w/v) and laponite‐RD 1.5% (w/v) (ratio 2:1:1 (w/w)), inset scale bar 1 mm.

Next, the extrusion behavior of the biomaterial inks was visually studied using pneumatic‐driven extrusion of the biomaterial inks through a tapered conical nozzle (G25, ID 250 µm) (**Figure** **4** and Videos S1–S11, Supporting Information). This type of experiment gives additional insight into the flow behavior of the biomaterial inks compared to rheological experiments.^[^ ^46^, ^48^ ^]^ To visually assess the extrusion behavior of the different biomaterial inks, the inks were extruded midair at increased pneumatic pressure until continuous extrusion is achieved as shown in Figure 4. The flow behavior of the biomaterial inks was determined by interplay of several forces: i) gravity, which extends the extruded biomaterial ink; ii) surface tension, which forces the extruded biomaterial ink to form a droplet; iii) viscosity, which slows down the extension of the biomaterial ink under influence of gravity and formation of a droplet under the influence of surface tension; and iv) elastic behavior, which opposes the formation of droplet and extension of the biomaterial ink. The formation of droplets rather than continuous filaments is a sign of a strong contribution of surface tension on the extruded biomaterial ink – surface tension can generate sufficient forces to deform the material on the time scale of extrusion. Hence, droplet‐like formation would result in poor shape‐fidelity of the 3D printed structure without any additional support.^[^ ^1^ ^]^

Alginate in water and media shows a droplet‐like formation at the tip of the needle with no continuous filament or strand formation (Figure 4a; Videos S1 and S2, Supporting Information).^[^ ^8^ ^]^ This means that the material undergoes viscous flow rather than stress‐induced (plastic) flow. Whereas pure SCMC in water and in media as well as laponite in water formed a continuous filament (Figure 4b,d; Videos S3, S4, and S7, Supporting Information). It is also interesting that the character of the formed filament is different, in which laponite forms a smooth filament, while SCMC‐blends form a “rough” filament that may be an indication of some inhomogeneity of the material. Likewise, the blending of alginate with SCMC results in the formation of a filament in water and in media (Figure 4c, Videos S5 and S6, Supporting Information).^[^ ^8^ ^]^ This means that these biomaterial inks flow with the dominant contribution of flow stress (plastic behavior), in which the flow stress is higher than the surface tension force. Interestingly, this flow stress behavior was not observed in rotational rheology experiments, which may be due to that the value of flow stress is lower than the measured range of stress in rheology experiments. The continuous filament formation is an indication of gelation or increase in viscosity at low stress due to reestablishing contacts and or non‐covalent bonds formation between particles or polymer chains. This filament‐like flow is crucial for maintaining the end structure shape fidelity after printing.^[^ ^8^, ^11^, ^46^, ^48^ ^]^ While laponite forms a continuous filament in water, laponite blended with alginate in water and in media forms a filament with a pronounced droplet at the end (Figure 4e; Videos S8 and S9, Supporting Information). Blending alginate with laponite and SCMC results in the formation of filament with some thickening/rounding at the end of the tip of the filament (Figure 4f; Videos S10 and S11, Supporting Information). Both of these inks are opaque, while pure laponite in water is transparent. These observations are an indication that the negatively charged alginate reduces interactions between laponite particles. Whereas interactions between SCMC polymer chains are not influenced by alginate. In addition to that, the addition of laponite and SCMC enhances the extrusion properties of alginate from droplet‐like to filament‐like extrusion behavior, which is also in good relation to the rheological results presented previously.

Figure 4 shows the mass flow of the biomaterial inks at different extrusion periods for the assessment of the consistency in the material volumetric flow through extrusion. For this, a relatively small amount of material was extruded in order not to change the conditions of the experiments. In these experiments, the applied shear stress (*τ*) is the ratio of applied force to area of biomaterial ink in contact with the dispenser, *F*  =  *P* × *A*, where *P* is the pneumatic pressure and *A* is the surface cross‐sectional area of the biomaterial ink in contact with pressurized air that is the cross‐sectional area of the dispenser of the 3D (bio) printing cartridge, *A*  =  π*r* ^2^. Extrusion of considerable amounts of materials results in the decrease of contact area with the dispenser ( *A_ink_* =  2π*rh*, where h is the height of the biomaterial ink in the dispenser and r is the inner radius of the dispenser). Hence it results in an increase in shear stress ($\tau \approx \frac{P\pi r^{2}}{2\pi rh}\approx \frac{Pr}{2h}\approx \frac{1}{h}$), and in turn it results in faster flow – extrusion out of nearly empty syringe is considerably easier than that out of a dispenser full of biomaterial ink. All formulations except for laponite‐based blends have shown a linear flow of extruded mass of material with increasing extrusion time. Such kind of behavior is expected for systems, in which viscosity recovery remains constant during the experiment and there is no thixotropic behavior. On the other hand, laponite‐based blends show nonlinear mass flow with time. As discussed earlier, these blends exhibit thixotropic behavior, when incomplete recovery of their viscosity after high shear stress is observed. Thus, the reason for nonlinear flow behavior is the structural changes in material such as the breaking of contacts between laponite particles that results in the decrease of viscosity with time under applied pressure.

Summing up rheological properties of inks in cell culture media, which are important for further discussion about the behavior of cells; alginate is less viscous and demonstrates pure viscous behavior, alginate with laponite (laponite forms aggregates) has nearly the same rheological as pure alginate. On the contrary, pristine SCMC and alginate blended with SCMC are more viscous, and they demonstrate flow stress and shear thinning behavior. A blend of alginate, SCMC, and laponite is an intermediate between alginate and alginate blended with SCMC.

**Figure 5:**
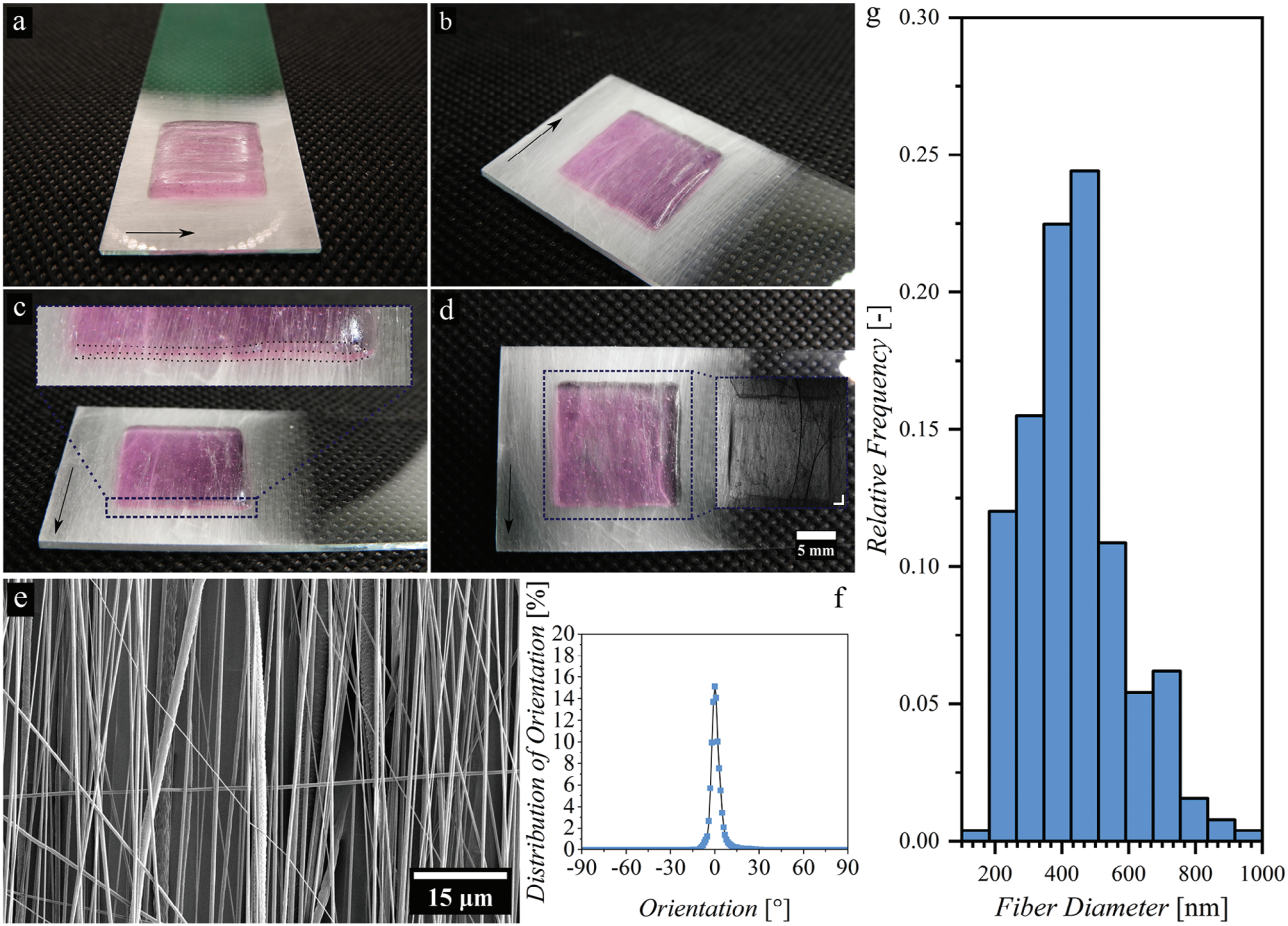
The 3D printed – touch‐spun multilayered construct at different views, where the arrows indicate the main fiber direction, a–c) perspective view, small dots in inset show the different layer boundaries and d) top view with microscopic view in the inset (scale bar 2 mm) and fiber characterization with e) scanning electron microscope (SEM) image of the touch‐spun fibers, f) fiber orientation plot, and g) fiber diameter distribution.

Next, we fabricated a multilayered construct by sequential extrusion 3D (bio) printing of bioinks (cells + biomaterials) and touch spinning of PCL nanofibers to study fibroblast behavior within this structure (Figure 1 and **Figure** **5**). For the preparation of the sequential multilayered structure, the process starts with the touch‐spinning of PCL nanofibers followed by 3D (bio) printing of the bioink (1 layer = nanofibers + bioink), in which this layer was repeated three times with additional touch‐spun nanofibers layer at the top of the 3‐layer construct to form a “sandwich‐like” structure (top and bottom are nanofibers). The average diameter of the nanofibers is 436 ± 148 nm (Figure 5g), and the average density of the fiber layers is 35 ± 8%. Additionally, SEM images of the PCL touch‐spun nanofibers with the distribution of orientation of the fibers is shown in Figure 5e,f, in which the nanofibers have shown an average alignment of 85 ± 3%. In the end, this approach allows for the hands‐free fabrication of multilayered cell‐laden constructs (bioinks) combined with highly aligned fibers in different geometries and within a cell‐friendly environment. This will eliminate the need for the multistep incorporation of bioinks post‐printing into already fabricated fibrous constructs usually seen in most commonly used fiber fabrication methods such as electrospinning and MEW.

Finally, we studied the effect of the rheological properties of bioinks on cell viability and interactions between fibroblast cells and the touch‐spun PCL nanofibers within the fabricated multilayered bioink‐nanofibrous constructs. Fibroblasts viability in the pristine bioink with and without touch‐spun PCL nanofibers was evaluated (Figures S24 and S25, Supporting Information). All samples have shown high cell viability of higher than 80% at day 7 of incubation with a general increase in viability between days 3 and 7. Alginate with fibers shows an increase in viability from 83 ± 5% at day 3 of incubation to 93 ± 3% at day 7 of incubation. Additionally, the viability of cells in the bioinks with fibers is slightly lower (non‐significant) compared to pristine bioinks and the control. For instance, viability of 90 ± 4% was observed for SCMC bioink with fibers compared to 95 ± 2% without fibers on day 7 of incubation. This can be explained by the reduced cell viability in the case of PCL touch spun nanofibers alone, which is 86 ± 4 and 81 ± 4% for day 3 and day 7, respectively. Thus, fibers may have a negative effect on cell viability in the first days of incubation if highly viscous inks with flow stress behavior are used. With time cell viability becomes independent of ink properties and the presence of fibers. The obtained cell viability in the composite constructs fabricated using this approach is comparable to composites fabricated by other techniques such as MEW^[^ ^2^, ^5^ ^]^ and electrospinning,^[^ ^7^, ^14^, ^30^, ^31^ ^]^ although usually in these techniques fibrous mats are added post‐printing. Thus far, it can be concluded that all formulations have shown high cell viability with no significant effect of PCL touch‐spun fibers on the overall viability of fibroblasts in the construct.

**Figure 6:**
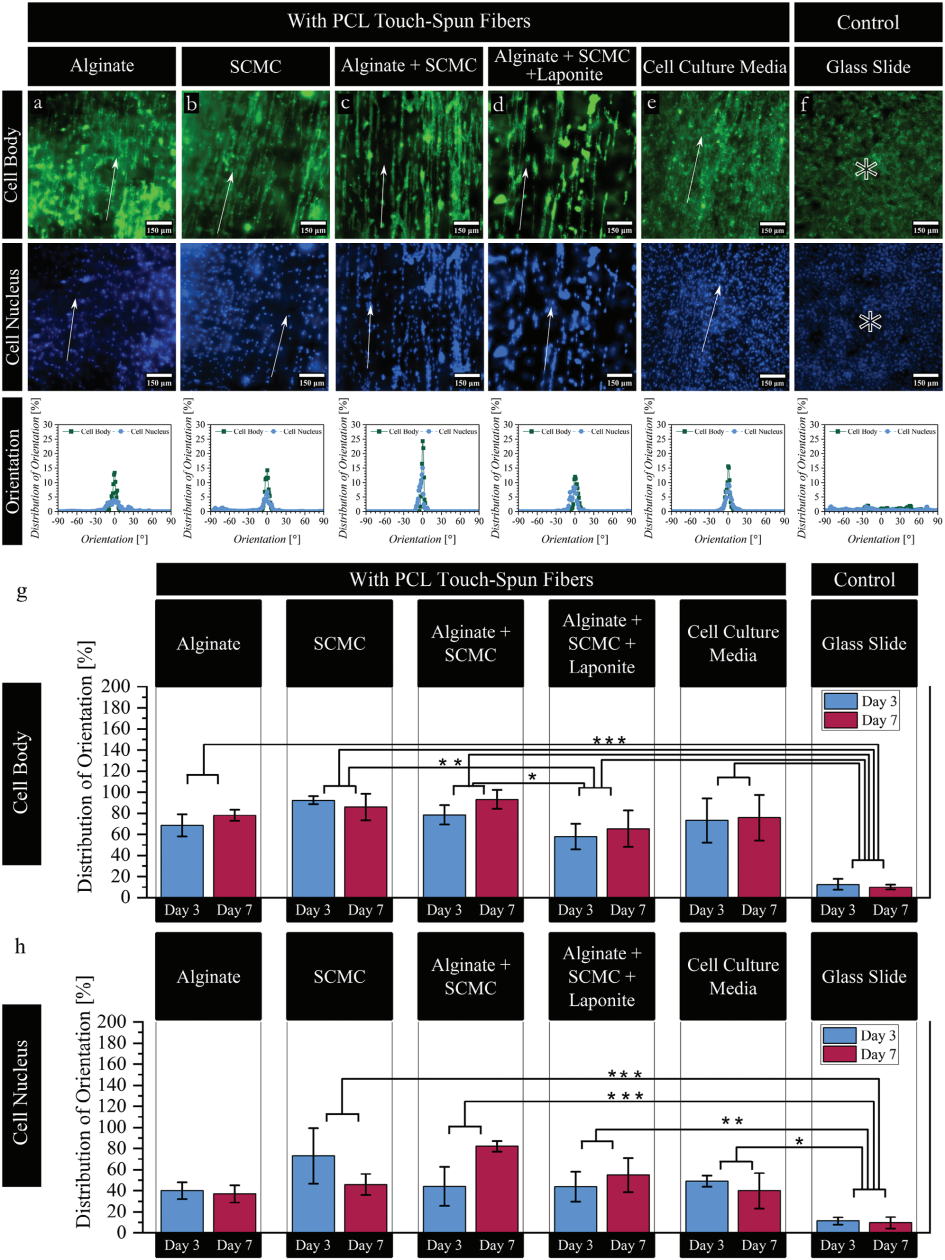
Fibroblasts orientation within the multilayered 3D bioprinted bioink – PCL touch‐spun fibers construct, multilayered PCL touch‐spun fibers (cells suspended in cell culture media), and control (cells suspended in cell culture media and directly seeded on a glass slide), where a–f) is the fibroblasts orientation at day 7 of incubation and g,h) alignment degree at days 3 and 7 of incubation. Green is the cell body (actin filaments), blue is the cell nucleus, and arrows indicate the main direction of fibers (0°). Fluorescence microscopy images were taken at 20× magnification, and the scale bar is 150 µm. Alignment degree is measured within ±5° around the main direction 0° (mean ± SD * *p* ≤ 0.05, ** *p* ≤ 0.01, *** *p* ≤ 0.001. *n* = 3).

We further studied the effect of the bioink material on the ability of fibroblast cells to align along the PCL touch‐spun nanofiber's main direction. Namely, pristine alginate, pristine SCMC, a mixture of alginate and SCMC as well as a mixture of alginate, SCMC, and laponite were tested. Pristine laponite (was not able to form a gel) and alginate mixed with laponite (frequent clogging, Figure 4e) were not studied. Fibroblasts (suspended in cell culture media) seeded on a glass substrate were used as a negative control sample (**Figure** **6**, Figures S26 and S27, Supporting Information). Generally, fibroblasts show a preferred orientation along the nanofiber main direction in bioinks used compared to the control (glass substrate), where a random distribution of orientation was observed. In addition to that, all bioinks studied allow a high alignment degree of the cell body of higher than 60% whereas cell nuclei have shown a relatively lower degree of alignment of higher than ≈40%. It was also observed that bioinks have an effect not only on the degree of nuclei alignment but also on their shape. For instance, nuclei of fibroblasts seeded within touch‐spun PCL fibers have shown an average aspect ratio of 1.8 ± 0.3 and an average circularity of 0.8 ± 0.1. Additionally, fibroblasts nuclei have shown an average aspect ratio of 2.1 ± 0.6 and an average circularity of 0.8 ± 0.1 in alginate + SCMC with PCL fibers construct. This can be observed in the nuclei shape in Figure S27, Supporting Information.

**Figure 7:**
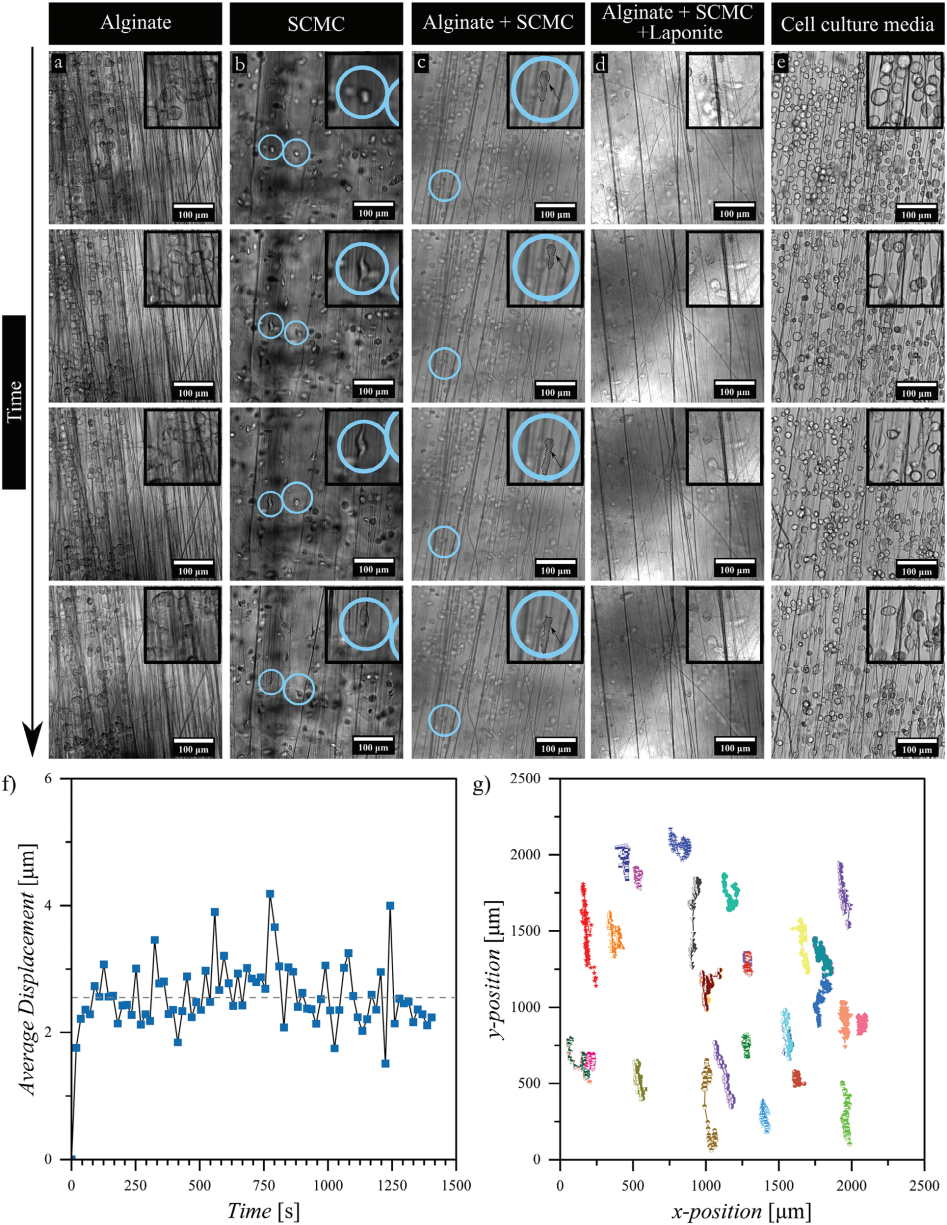
Time‐lapse (24 h) of fibroblast cells within a–d) multilayered 3D bioprinted bioink – PCL touch‐spun fibers constructs and e) multilayered PCL touch‐spun fibers construct, as well as live tracking of fibroblasts cells, where f) average displacement versus time profile and g) spatiotemporal displacement showing cell trajectories of the cells suspended in cell culture media and cultured between PCL touch‐spun layers. The inset shows a close‐up view of a few cells in an area of interest in the images, the scale bar is 100 µm.

We quantified the effect of the bioink on the degree of cell alignment. Cell body in the 3D bioprinted alginate with fibers show an alignment degree of 70 ± 10 and 79 ± 5% at days 3 and 7, respectively, indicating an increase in alignment of ≈10% (Figure 6a,g,h). Whereas SCMC with touch‐spun fibers showed a relatively high degree of cell body orientation (around an angle of ±5°) of 92 ± 4 and 86 ± 13% on days 3 and 7, respectively (Figure 6b,g,h). Similarly, fibroblasts suspended in pure cell culture media and seeded between PCL touch spun nanofibrous layers show a preferred orientation of their body with a relatively lower degree of cell body alignment of 73 ± 21 and 76 ± 21% at days 3 and 7 of incubation, respectively (Figure 6f,g,h). Additionally, alginate blended with SCMC show the highest degree of alignment of 78 ± 9 and 93 ± 9 at day 3 and 7 indicating an increase of about 15% from day 3 to 7 of incubation (Figure 6c,g,h). Alginate blended SCMC and laponite shows the lowest degree of alignment of 58 ± 12 and 65 ± 17% on days 3 and 7 (Figure 6d,g,h). In this way, the addition of laponite, which increases viscosity and introduces flow‐stress plastic behavior, results in a lower degree of cell alignment and this can be also observed in the cell motility results, which fibroblasts showed minimal cell spreading within the time frame studied of 24 h (**Figure** **7**d and Video S15, Supporting Information). Interestingly, alginate + SCMC, which its solution has the highest viscosity and pronounced shear thinning, allows the highest degree of cell alignment, which is higher than that observed in alginate with zero‐shear viscosity and with pure cell culture media with low water‐like viscosity. On the other hand, random orientation of fibroblasts with a low degree of alignment is observed in the control sample (glass slide), in which the alignment degree is significantly lower (13 ± 5 and 10 ± 2% at days 3 and 7, respectively) in comparison to PCL nanofibers containing samples (Figure 6a–e,g,h). Thus far, it can be concluded that fibroblasts exhibit a preferred orientation with a relatively high degree of alignment in the multilayered 3D printed bioink – PCL touch‐spun nanofibrous constructs.

It is also important and noteworthy to mention that bioink materials have an essential effect on the formation of agglomerates of cells. No cell agglomerates were observed when pure cell culture media was used to suspend the cells. On the other hand, a small degree of agglomeration was observed in pure alginate, SCMC, and alginate mixed with SCMC. Whereas very large agglomerates were observed in the alginate + SCMC + laponite mixture. The reason for this cell clustering is the different rheological/mechanical properties of the biomaterial inks. Cell culture media is less viscous, in which its viscosity is close to that of pure water which allows for easy dispersion of cells. The viscosity of alginate and SCMC is much higher which hinders the dispersion of cells and as a result, some cell clusters remain. The addition of laponite in the case of alginate + SCMC + laponite results in very strong agglomeration of cells that is due to the complex ionic nature of laponite, in which laponite crystals have negatively charged faces and positively charged edges that are sensitive to pH variations, which in water it results in aggregation of laponite particles formation of colloidal gel.^[^ ^10^, ^44^ ^]^ Additionally, laponite has shown its ability to interact with a variety of molecules and charged particles.^[^ ^9^, ^10^ ^]^

Finally, we investigated the effect of rheological properties of inks on cell motility within the fabricated constructs as shown in Figure 7 and Videos S12–S16, Supporting Information. A considerable number of fibroblasts in the alginate ink (Figure 7a and Video S12, Supporting Information) were able to sense, adhere to, and move along the fibers. However, the cells remained mostly rounded and their movement was rather an oscillation around the same point. In SCMC and alginate blended with SCMC, the number of adhered fibroblasts was smaller, although some of them were able to travel over a distance comparable to their own size with elongation of their body (Figure 7b,c; Videos S13 and S14, Supporting Information). Furthermore, we speculate that the addition of laponite – observed as agglomerates of small particles – completely hindered fibroblast's motility within the hydrogel (Figure 7d and Video S15, Supporting Information) although rheological studies showed that this ink is able to flow and its viscosity is comparable to that of alginate and alginate blended with SCMC. The highest cell density and motility were observed in pure cell culture media with a quarter of the total number of cells (*n* = 394) attached to the fibers. In addition to that the attached cells were able to glide along the fibers, change their morphology from rounded to spindle‐like structure (elongated), and snap back and forth from the fibers and jump between them (Figure 7e and Video S16, Supporting Information). Furthermore, cells suspended in cell culture media and cultured between fiber layers presented an average displacement of 3.5 µm⋅s^−1^ with a directed movement along the fibers as demonstrated in Figure 7f,g. It is noteworthy that the fibroblasts motility was studied directly after 3D (bio) printing for a total duration of 24 h.

## 3 Conclusion

In conclusion, we for the first time demonstrate the hands‐free biofabrication of multilayered bioink – nanofiber composite construct using a custom‐made 3D (bio) printer with integrated touch spinning setup and investigated the effect of inks on the interaction of fibroblast cells with the nanofibers. The rheological properties of alginate‐based biomaterial inks were thoroughly studied to understand their viscoelastic and flow properties in both water and fibroblast cell culture media. The pure alginate biomaterial ink shows zero‐shear viscosity, whereas alginate with laponite (laponite forms aggregates) has nearly the same rheological properties as pure alginate. On the other hand, SCMC and alginate blended with SCMC are the most viscous and demonstrate flow stress and shear thinning behavior. On the contrary, the rheological properties of the blend of alginate with SCMC and laponite are intermediate between alginate and alginate blended with SCMC. Biomaterial inks based on laponite are highly sensitive to the ionic strength of the solvent and even hinder its thixotropic properties. Fibroblasts demonstrated high cell viability on the fibers and were able to align along the fiber's direction in all kinds of inks used in this study although the increase of viscosity and in particular, the addition of laponite resulted in strong agglomeration of cells. The rheological properties of the biomaterial inks were demonstrated to have a considerable effect on the ability of cells to adhere to the fibers and move along them. The highest motility was observed in less viscous cell culture media, increased viscosity reduced the motility of fibroblasts although they were still able to oscillate on the same spot and in some instances moving a considerable distance on the fibers. On the other hand, laponite completely hindered cell motility. The obtained knowledge in this study is highly important for the fabrication of tissues and in particular tissues with fibrous structures and uniaxial orientation of cells such as connective and muscle tissues. Moreover, we believe that this integrated biofabrication method would allow for a simple, hands‐free, well‐controllable, and biologically compatible environment for the fabrication of bioink‐nanofiber composite without the need for extremely high voltages cutting the limitations of current fabrication methods such as electrospinning and melt electrowriting.

## 4 Experimental Section

### Materials

DAPI BioChemica (4′,6‐diamidino‐2‐phenylindole, C_16_H_15_N_5_⋅2HCl, *M_w_* = 350.25 g⋅mol^−1^) was purchased from AppliChem, Darmstadt, Germany. LAPONITE‐RD (silicic acid lithium magnesium sodium salt, bulk density 1000 kg⋅m^−3^) was provided by BYK‐Chemie GmbH, Wesel, Germany. Alginic acid sodium salt (for biochemistry, *M_w_* = 300–350 kDa) and formaldehyde solution 37% (CH_2_O) (≥37%, for synthesis) were purchased from Carl Roth GmbH + Co. KG, Karlsruhe, Germany. Phalloidin DyLight 488 conjugated 300 (300 units⋅mL^−1^) was purchased from Cell Signaling Technology Europe, B.V. Leiden, The Netherlands. Dulbecco's Modified Eagle Medium (DMEM) (with 4.5 g⋅L^−1^ glucose, without L‐glutamine and sodium pyruvate with 3.7 g⋅L^−1^ NaHCO_3_ was purchased from PAN‐Biotech GmbH, Aidenbach, Germany. Dulbecco's Phosphate Buffered Saline (DPBS) (Modified, without calcium chloride and magnesium chloride), Ethanol absolute (ACS grade reagent, ≥99.5% (GC)), Gentamicin (10 mg⋅mL^−1^ in deionized water, BioReagent), Polycaprolactone ( ${\bar{M}}_{n}=\;$80 kDa, (C_6_H_10_O_2_)_n_), Sodium Carboxymethyl Cellulose (${\bar{M}}_{w}\;$≈ 700 kDa), and Triton X‐100 ( ${\bar{M}}_{n}=\;$625 g⋅mol^−1^, for molecular biology) were purchased from Sigma‐Aldrich Co Ltd., St. Louis, Missouri, USA. Calcein AM (C_46_H_46_N_2_O_23_, *M_w_* = 994.87), Ethidium Homodimer‐1 (EthD‐1) (C_46_H_50_Cl_4_N_8_, *M_w_* = 856.77), Fetal Bovine Serum (FBS) (qualified, Standard), GlutaMax Supplement 100X, and Penicillin‐Streptomycin 100X (5000 U⋅mL^−1^), were purchased from Thermo Fisher Scientific Inc., Waltham, Massachusetts, USA.

### Solutions Preparation

All the biomaterial inks were prepared in both sterilized distilled water and fibroblasts cell culture media. For this alginate 3.5%, SCMC 3%, and blended alginate 3% with SCMC 3% *w/v* were dissolved separately with a constant stirring of ≈150 rpm overnight. For the preparation of alginate blended SCMC, the solvents were blended in a dry state and then dissolved as described previously. Whereas, for the preparation of laponite 5% and laponite blends, first the solvent was stirred at 1000 rpm to form a vortex and laponite was added gradually to the solvent under constant stirring. Next, the solution was stirred at 1000 rpm for 20 min until laponite is completely dissolved and a transparent or clear solution is observed. Finally, pristine laponite is kept overnight to relax and for the blended solutions, either alginate alone or alginate mixed with SCMC was added to the already prepared laponite solution under a constant stirring of 200 rpm. For the preparation of the touch‐spinning solution, PCL 80 kDa was dissolved in chloroform with a final concentration of 10% *w/v*.

### 3D (Bio)Printing Integrated Touch Spinning Setup

The custom‐made setup was composed of a multi‐head unit with a vertical movement in the z‐axis and a platform, which moves in the x‐ and y‐axes as for conventional 3D printers. The setup was also composed of a touch‐spinning unit that is mounted on the platform described earlier. Additionally, the touch spinning unit was comprised of a rotating disk with two rods, where the extruded polymer solution droplet touches the rods while rotating for mechanically drawing fibers and depositing them. Furthermore, the setup had a stationary substrate/collector holder (e.g., for attaching a glass slide), where sequential 3D (bio) printing – touch spinning was performed to achieve an alternating layer‐by‐layer biofabrication of a construct comprising of alternating bioink – nanofibers layers.

### Samples Preparation

The samples were prepared by sequential 3D (bio) printing of bioinks and touch spinning of PCL fibers. For this, a custom‐made device was used as described earlier. For the preparation of the sequential multilayered structure, the process starts with touch‐spinning followed by 3D (bio) printing, in which it is repeated three times with an additional touch‐spun nanofibers layer at the top. For touch spinning PCL 10% (*w/v*) in chloroform was used as a spinning dope. The PCL solution was extruded through a 22 G straight metallic needle with a needle length of 13 mm. A lateral movement rate of 50 mm⋅min^−1^ of the touch‐spinning extrusion head was applied along the rotating rods (along the y‐axis; perpendicular to the axis of rotation) to cover a lateral distance of 18 mm. Furthermore, a rotational speed of ≈3000 rpm was used to mechanically draw PCL fibers and deposit them. The 3D (bio) printed layer size was set to 15 × 15 mm^2^ with a printing speed of 200 mm⋅min^−1^ and with a 1 mm distance between printed strands. The biomaterial inks were 3D (bio) printed using a 25G (250 µm inner diameter) PTFE red tapered nozzle with a pneumatic pressure of 0.4, 0.5, 0.15, 0.8, 0.8, and 0.7 bar for alginate, SCMC, laponite, alginate with SCMC and laponite, alginate with SCMC, and alginate with laponite, respectively. In addition to that, the layers were printed in an alternating pattern (“woodpile”‐like structure).

### Rheological Characterization

The modular rheometer (Anton Paar MCR 702 MultiDrive Rheometer, Anton Paar GmbH, Graz, Austria) was used to study the rheological characteristics of the pristine hydrogels and their blends. For the rheological experiments; amplitude sweep, frequency sweep, thixotropy, creep‐recovery, and stress‐relaxation; a parallel plate geometry with a measuring diameter (*Ø*) of 25.01 mm and a gap distance of 0.60 mm in oscillatory mode was used. The flow experiments were operated using a cone‐plate geometry with a cone angle (*φ_o_*) of 0.93°, measuring diameter (*Ø*) of 59.97 mm, and a gap distance of 0.12 mm. Each experiment was carried out using hydrogel materials dissolved in water and in fibroblasts cell culture media. Additionally, 15 min have been waited before each measurement after the trimming step. This was done to allow for the relaxation or recovery of the material structure or to cancel out any instabilities in the structure of the material generated from the preparation and handling of the sample. Further, the poly dimethyl siloxane oil (silicone oil) was applied to the edges of the sample at the trimming position as well as around the measuring geometry to reduce the evaporation of the sample while conducting the measurements.

Amplitude sweep was conducted to detect the linear viscoelastic (LVE) region or limit as well as the flow point of the samples. For this, the experiment was operated at a constant oscillation frequency of 1 Hz and shear strain from 0.01 to 1000% with a ramp logarithmic profile for 25 data points. Next frequency sweep was done in a frequency range from 100 to 0.1 Hz with a ramp logarithmic profile at constant shear strain or deformation of 0.1% for 25 data points. For the stress‐relaxation experiments, the experiment was carried out at a constant shear strain of 1 and 10% for 1000 s with a ramp logarithmic data acquisition profile (0.01–30 s) for 264 data points. In addition to that, creep‐recovery experiments were executed at two‐ and three‐interval stress modes with a ramp logarithmic data acquisition mode (0.01–25 s). For the two‐interval creep test, the first interval was done at a constant stress of 10 Pa for 300 s and 91 data points. Whereas the second step was conducted at a constant stress of 0 Pa for 600 s and 185 data points. The three‐step or three‐interval creep test (3ITT) was performed at a constant shear stress of 10, 100, and 10 Pa for the first, second, and third intervals, respectively, and each for 91 data points. For the pristine laponite the second step was performed at a constant stress of 200 Pa since the applied stress of 100 Pa was not high enough to disturb the laponite structure. For thixotropy measurements, two modes were operated: oscillation three interval test (3ITT) and cyclic (3 cycles) test at a constant frequency of 1 Hz. For the 3ITT test, the first step was performed at 0.1% shear strain for 600 s, the second step at 1000% for 60 s, and the third step at 0.1% for 60 min. On the other hand, the cyclic test was carried out for 3 cycles at 0.1% and 1000% for the first and second intervals (both intervals were cycled three times in an alternating manner), respectively, for a duration of 120 s each and ended up with a 0.1% interval for 120 s.

Two flow experiments were performed: steady‐state shear rate ramp and shear rate ramp up‐constant‐down. The steady‐state test was conducted with an initial shear rate of 0.01 s^−1^ and a final shear rate of 1000 s^−1^ in a ramp logarithmic profile for an interval of 1024 s. The data acquisition profile was ramp logarithmic with an initial measuring duration of 100 s and a final duration of 0.05 s for 75 data points. For the ramp up‐constant‐down, a pre‐shear of a constant shear rate of 5 s^−1^ was performed for 30 s initially. The ramp was done from 5 to 131 s^−1^ (ramp up) and then kept constant at 131 s^−1^ for 180 s (constant) taking a data point each 3.5 s. Finally, a flow back from 131 to 5 s^−1^. The measuring interval for ramp up and down was 300 s taking a data point each 2.5 s (constant profile).

### Extrusion Test

Equal volumes of the different biomaterial inks were loaded into a 5cc syringe with a 25G (250 µm inner diameter) PTFE red tapered nozzle attached to it. Then the pressure was increased gradually until a continuous extrusion of the material was achieved. After that images and videos of the extruded biomaterial ink were taken at the tip of the nozzle.

### Mass Flow Determination

The loaded biomaterial inks were extruded at a selected pressure where the material extrudes continuously, and needle clogging is minimized. For this, the biomaterial inks were extruded at a pressure of 0.4, 0.5, 0.15, 0.8, 0.8, and 0.7 bar for alginate, SCMC, laponite, alginate with SCMC and laponite, alginate with SCMC, and alginate with laponite, respectively. The extruded material was collected on a weighing tray and the weight was recorded at different time intervals; 20, 30, 40, 50, and 60 s. For each time interval, three repeats were done, and the mean and standard deviation were calculated.

### Bioink Preparation

For the bioink preparation a suspension of BALB/3T3 fibroblasts at a cell density of 60 million cells⋅mL^−1^ were prepared in cell culture media supplemented with fetal bovine serum, GlutaMax, gentamicin, and Penicillin‐Streptomycin. First, the biomaterial inks were prepared in sterilized and distilled water as described above in the section (Solutions preparation) considering the additional liquid volume added by cell suspension to yield the desired final concentration. Subsequently, the cell suspension was added to the prepared biomaterial ink and stirred gently for 5 min at room temperature, until the cell suspension was mixed thoroughly. Finally, 2 mL of the bioink was transferred carefully under aseptic conditions into a sterile 5 cc (bio) printing cartridge with a plunger and an end cap to keep the bioink in sterile conditions while transferring the cartridge to the (bio) printing integrated touch‐spinning setup.

### SEM Analysis of the Touch‐Spun Fibers

The microscopic properties of the touch‐spun nanofibers were investigated using the VolumeScope scanning electron microscope (SEM) (Thermo Fisher Scientific GmbH, Germany). The images were analyzed using ImageJ 1.53 (Wayne Rasband and contributors, National Institutes of Health, USA).^[^ ^49^ ^]^ For the measurement of the fiber diameter distribution, several images taken at 15 000× magnification were analyzed using the line tool of ImageJ with a total measurement of 259. Whereas for the measurement of fiber layer density, images were taken at 15 000× and 5000× magnification and the density was calculated as the total area of the fibers to the total area of the image. Fiber distribution of orientation was measured using the OrientationJ (version 19.11.2012 – Biomedical Imaging Group (BIG), Ecole Polytechnique Fédérale de Lausanne (EPFL) Lausanne, Switzerland) plugin of ImageJ.^[^ ^49^ ^]^ For that, the cubic spline gradient method with a Gaussian window σ of 10 pixels and minimum coherency and energy of 0% was used. Additionally, the alignment degree was calculated by taking the distribution of orientation at ±5° around the 0° direction over the total distribution of orientation.

### Actin Filament (F‐Action) – Cell Nucleus Staining and Cell Orientation Determination

The alignment of BALB/3T3 fibroblasts was studied by visualizing the actin filaments of the cell body using phalloidin and cell nucleus using DAPI staining solution at days 3 and 7 of incubation. The final staining solution is composed of 10 mL DPBS, 250 µL Phalloidin Dylight 488 (300 units mL^−1^ dissolved in methanol), and 10 µL DAPI (5 mg⋅mL^−1^ in dimethylformamide (DMF)). Initially, the fibroblasts cell culture media was aspirated, and then the samples were washed two times with DPBS for 5 min each. In the next step, the cells were fixed with formaldehyde (FA) 3.7% for 15 min and washed twice with DPBS to remove any residues of the fixative solution. After that, the cell membrane was permeabilized by 0.1% Triton 100 X solution in DPBS at a concentration of 1 µg⋅mL^−1^ for 5 min at ambient conditions and then washed twice with DPBS. Finally, the Phalloidin and DAPI‐containing staining solution was added to cover the samples entirely and incubated for 30 min at room temperature, and then the staining solution was aspirated and washed two times with DPBS. In the end, the samples were kept in DPBS and covered with aluminum foil before visualizing them under the Nikon Ti2 fluorescence microscope (Nikon Eclipse Ti2 inverted microscope, Nikon Instruments Inc., Tokyo, Japan). OrientationJ (version 19.11.2012 – Biomedical Imaging Group (BIG), Ecole Polytechnique Fédérale de Lausanne (EPFL) Lausanne, Switzerland), which is a plugin for ImageJ was used to detect the distribution of orientation in the fibroblasts cell body and nucleus, using the cubic spline gradient method with a Gaussian window of 100 pixels and minimum coherency and energy of 0%. Additionally, the alignment degree was calculated by taking the distribution of orientation at ±5° around the 0° direction over the total distribution of orientation.

### Live/Dead Assay

To determine the cell viability of the fabricated samples, a staining solution of 3.6 µL of Calcein AM (≈2 µm) and 4 µL (≈4 µm) of ethidium homodimer‐1 in 2 mL of DPBS was prepared. In which, Calcein AM was used to stain live cells, emitting a fluorescently detected green Calcein. Whereas, ethidium homodimer‐1 was used to stain dead cells, emitting a fluorescently detected orange fluorescence. For this, the prepared solution was added to the petri dish in a sufficient amount to entirely cover each sample and kept at room temperature for 30 min in dark conditions. Finally, the samples were visualized under the Nikon Ti2 microscope, the number of dead and live cells was counted from 5 images each, and the average cell viability was calculated on days 3 and 7.

### Live Cell Microscopy and Cell Tracking Analysis

Fibroblasts motility was assessed on the multilayered 3D bioprinted touch‐spun constructs (alginate, SCMC, alginate with SCMC and Laponite, and alginate with SCMC) as well as PCL touch spun fibers using the ND and the AVI/MP4 acquisition modes of the NIS‐Elements software of the Nikon Ti2 fluorescence microscope. For this, the constructs were fabricated as described previously and installed in the stage top incubator chamber with air, CO_2_, humidity, and temperature controllers (UNO STAGE TOP INCUBATOR, H301‐NIKON‐TI‐S‐ER xy‐motorized stage with 2GF‐MIXER, OKOLAB S.R.L., Naples, Italy). The temperature of the incubator was set to 37 °C with air and CO_2_ flow controlled to get 95% air and 5% CO_2_ as well as a humidity level of greater than 85%. Time‐lapse image sequence of the fibroblasts was acquired with a total duration of 24 h at 40× magnification brightfield. The acquired images were analyzed using Fiji/ImageJ.^[^ ^50^ ^]^ To identify individual cells, background shading and temporal drift corrections were performed using the ImageJ plugin BaSiC.^[^ ^51^ ^]^ Spatiotemporal position and displacement of a total of 30 cells were obtained with the Manual Tracking plug‐in for ImageJ (Fabrice Cordelières, Institut Curie, Orsay, France)).^[^ ^49^ ^]^

### Statistical Analysis

Data are represented as mean ± standard deviation (SD). Statistical analysis was done to examine the statistical significance of the tested samples and groups. Multiple comparisons of two‐way analysis of variance (two‐way ANOVA) followed by the multiple comparison test of the critical‐value type of the Tukey post‐hoc method was performed using the Origin 2022 SR1 9.9.0.225 (Academic) software (Origin, Version 2022, OriginLab Corporation, Northampton, MA, USA). The comparisons were considered significant, with *p < 0.05 as a significance level and the p‐values were presented as follows * *p* ≤ 0.05, ** *p* ≤ 0.01, *** *p* ≤ 0.001. A minimum number of samples of 3 (*n* = 3) was used for each variant.

## Conflict of Interest

The authors declare no conflict of interest.

## Supporting information

Supporting Information

Supplemental Video 1

Supplemental Video 2

Supplemental Video 3

Supplemental Video 4

Supplemental Video 5

Supplemental Video 6

Supplemental Video 7

Supplemental Video 8

Supplemental Video 9

Supplemental Video 10

Supplemental Video 11

Supplemental Video 12

Supplemental Video 13

Supplemental Video 14

Supplemental Video 15

Supplemental Video 16

## Data Availability

The data that support the findings of this study are available in the supplementary material of this article.
