# Supplementary material for: Biofabrication of Composite Bioink‐Nanofiber Constructs: Effect of Rheological Properties of Bioinks on 3D (Bio)Printing and Cells Interaction with Aligned Touch Spun Nanofibers
Source: Adv Healthc Mater. 2023 Nov 27;13(6):2303343. doi: 10.1002/adhm.202303343 (PMC11469018; doi:10.1002/adhm.202303343)
Supplement: Supplementary file 1 — Supporting Information [file ADHM-13-2303343-s006.pdf]

ADVANCED
HEALTHCARE
MATERIALS

Supporting Information

for *Adv. Healthcare Mater.*, DOI 10.1002/adhm.202303343

Biofabrication of Composite Bioink-Nanofiber Constructs: Effect of Rheological Properties of Bioinks on 3D (Bio)Printing and Cells Interaction with Aligned Touch Spun Nanofibers

*Waseem Kitana, Victoria Levario-Diaz, Elisabetta Ada Cavalcanti-Adam and Leonid Ionov**

Supporting Information

Biofabrication of Composite Bioink-Nanofiber Constructs: Effect of Rheological Properties of Bioinks on 3D (Bio) Printing and Cells Interaction with Aligned Touch Spun Nanofibers

*Waseem Kitana, Victoria Levario-Diaz, Elisabetta Ada Cavalcanti-Adam and Leonid Ionov**

Prof. Dr. L. Ionov and W. Kitana

Professorship of Biofabrication, Faculty of Engineering Science, University of Bayreuth, Ludwig-Thoma-Straße 36A, 95447 Bayreuth, Germany

Prof. Dr. Dr. E.A. Cavalcanti-Adam and Dr. V. Levario-Diaz

Department of Cellular Biophysics, Max Planck Institute for Medical Research, Jahnstraße 29, 69120 Heidelberg, Germany

Prof. Dr. Dr. E.A. Cavalcanti-Adam

Professorship of Cellular Biomechanics, Faculty of Engineering Science, University of Bayreuth, Universitätsstraße 30, 95447 Bayreuth, Germany

Prof. Dr. L. Ionov

Bavarian Polymer Institute, University of Bayreuth, Universitätsstraße 30, 95447 Bayreuth, Germany

*E-mail: leonid.ionov@uni-bayreuth.de

Oscillation experiments:

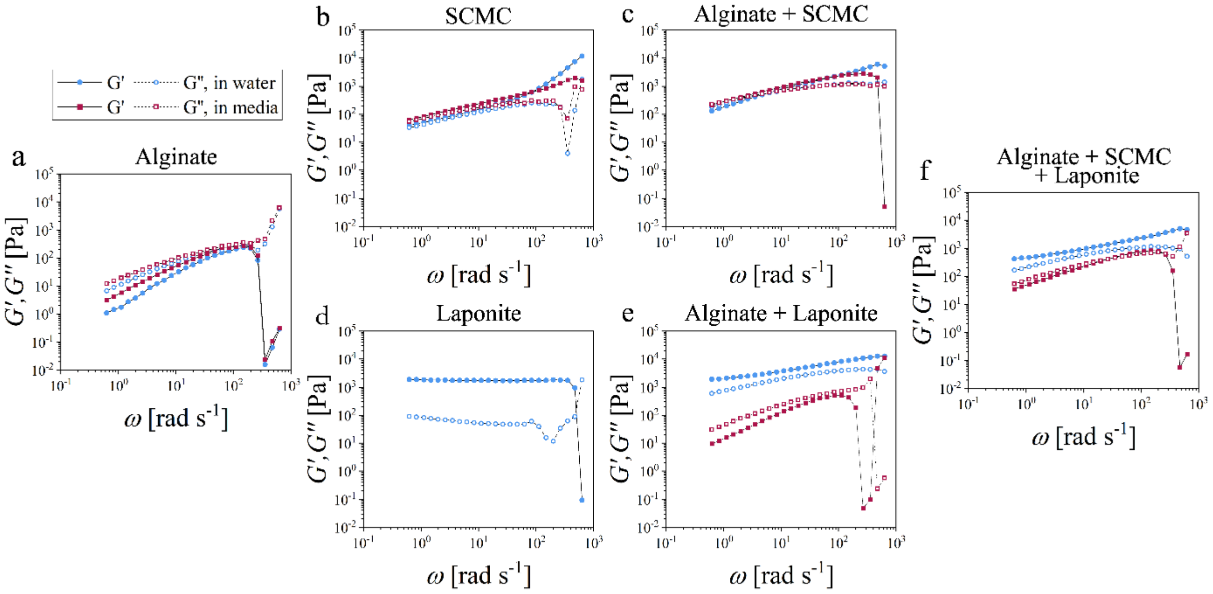

Figure S1. Frequency sweep (storage modulus (G') and loss modulus (G'') vs. angular frequency (ω)) in water and cell culture media for a) pristine alginate 3.5 % (*w/v*), b) pristine sodium carboxymethyl cellulose 3 % (*w/v*), c) blended alginate 3.5 % (*w/v*) with SCMC 3 % (*w/v*), d) pristine laponite-RD 5 % (*w/v*), e) blended alginate 3.5 % (*w/v*) with laponite 5 % (*w/v*), and f) blended alginate 3 % (*w/v*) with SCMC 3 % (*w/v*) and laponite-RD 1.5 % (*w/v*) (ratio 2:1:1 (*w/w*)).

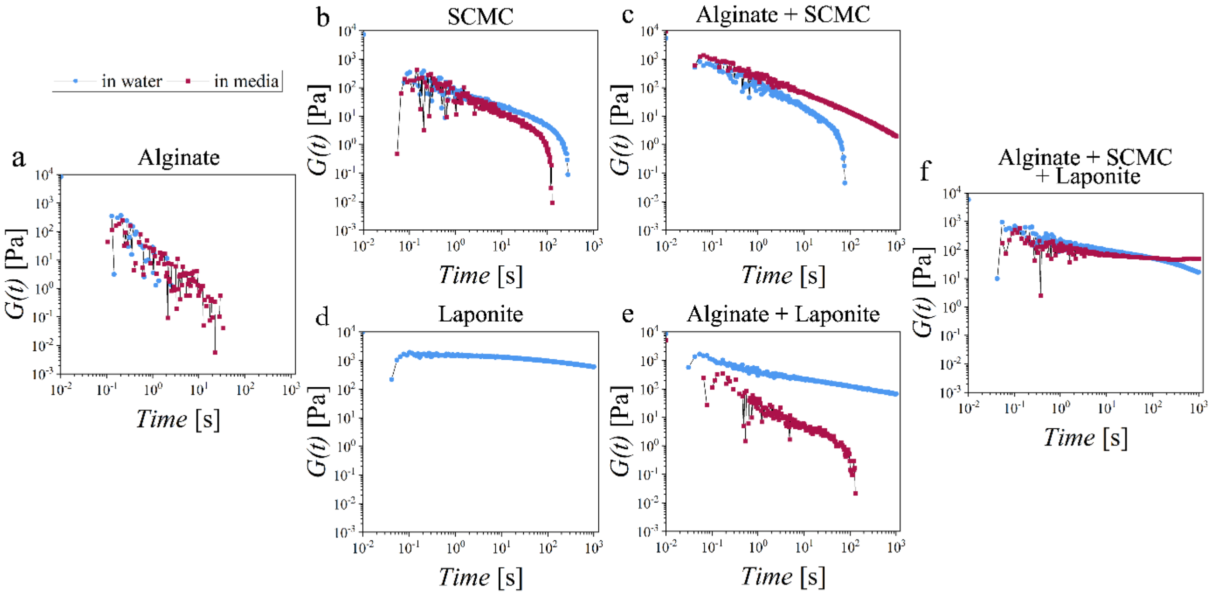

Figure S2. Stress relaxation modulus (G(t)) at 1 % shear strain in water and cell culture media for a) pristine alginate 3.5 % (*w/v*), b) pristine sodium carboxymethyl cellulose 3 % (*w/v*), c) blended alginate 3.5 % (*w/v*) with SCMC 3 % (*w/v*), d) pristine laponite-RD 5 % (*w/v*), e)

blended alginate 3.5 % (*w/v*) with laponite 5 % (*w/v*), and f) blended alginate 3 % (*w/v*) with SCMC 3 % (*w/v*) and laponite-RD 1.5 % (*w/v*) (ratio 2:1:1 (*w/w*)).

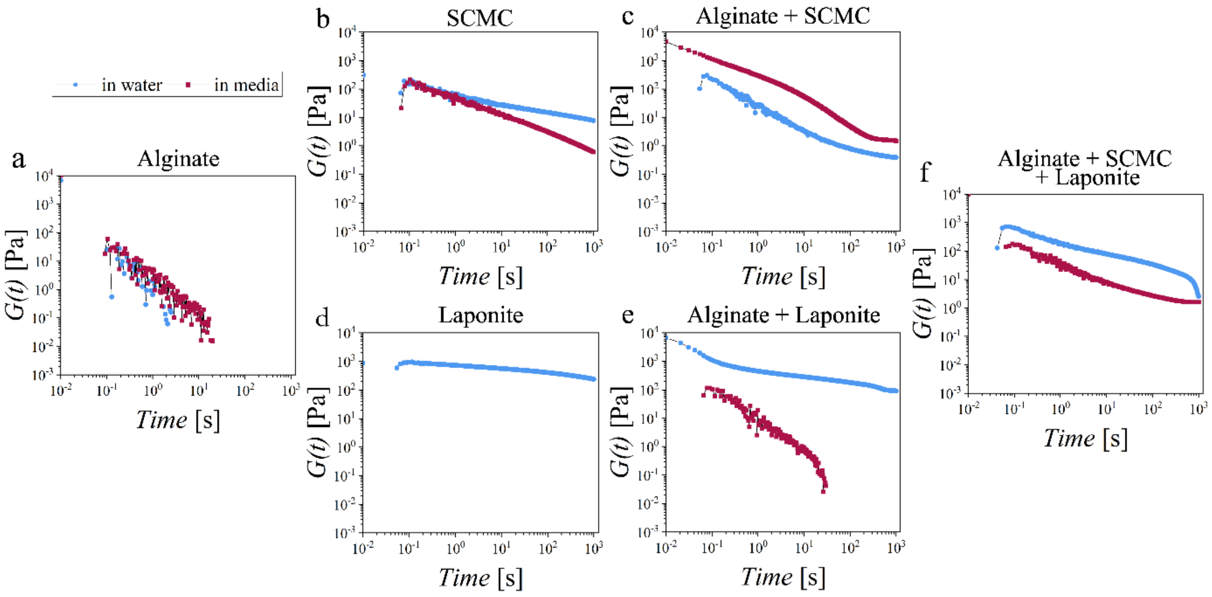

Figure S3. Stress relaxation modulus (G(t)) at 10 % strain shear strain in water and cell culture media for a) pristine alginate 3.5 % (*w/v*), b) pristine sodium carboxymethyl cellulose 3 % (*w/v*), c) blended alginate 3.5 % (*w/v*) with SCMC 3 % (*w/v*), d) pristine laponite-RD 5 % (*w/v*), e) blended alginate 3.5 % (*w/v*) with laponite 5 % (*w/v*), and f) blended alginate 3 % (*w/v*) with SCMC 3 % (*w/v*) and laponite-RD 1.5 % (*w/v*) (ratio 2:1:1 (*w/w*)).

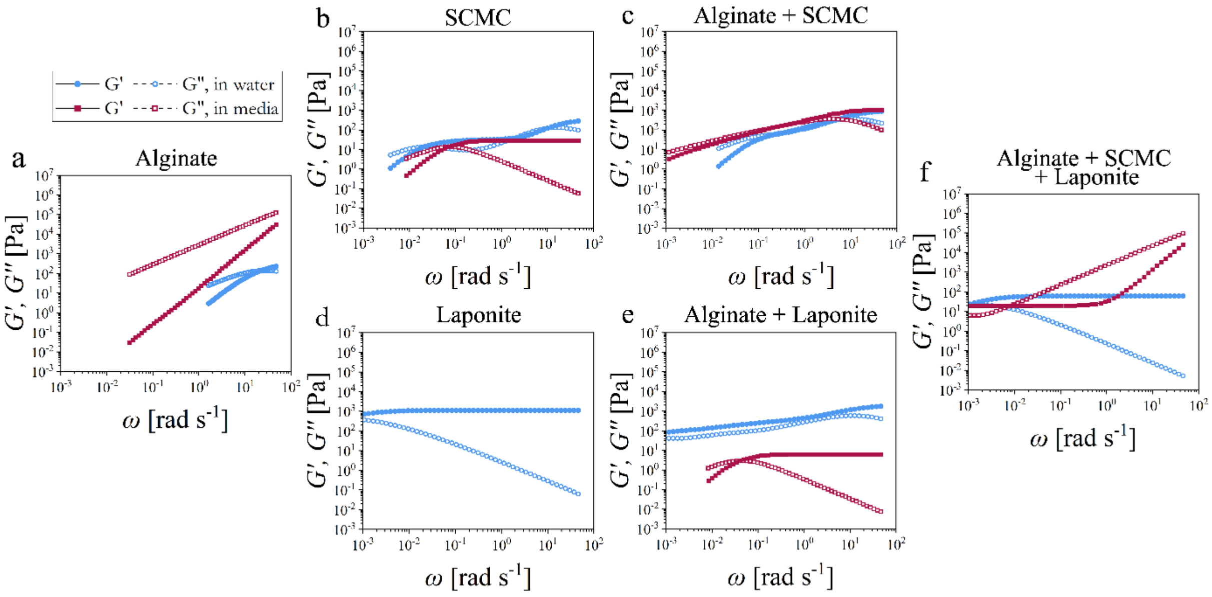

Figure S4. Frequency sweep from relaxation (storage modulus (G') and loss modulus (G'') vs. angular frequency (ω))at 1 % strain in water and cell culture media for a) pristine alginate 3.5 % (*w/v*), b) pristine sodium carboxymethyl cellulose 3 % (*w/v*), c) blended alginate 3.5 % (*w/v*) with SCMC 3 % (*w/v*), d) pristine laponite-RD 5 % (*w/v*), e) blended alginate 3.5 % (*w/v*) with

laponite 5 % (*w/v*), and f) blended alginate 3 % (*w/v*) with SCMC 1.5 % (*w/v*) and laponite-RD 1.5 % (*w/v*) (ratio 2:1:1 (*w/w*)).

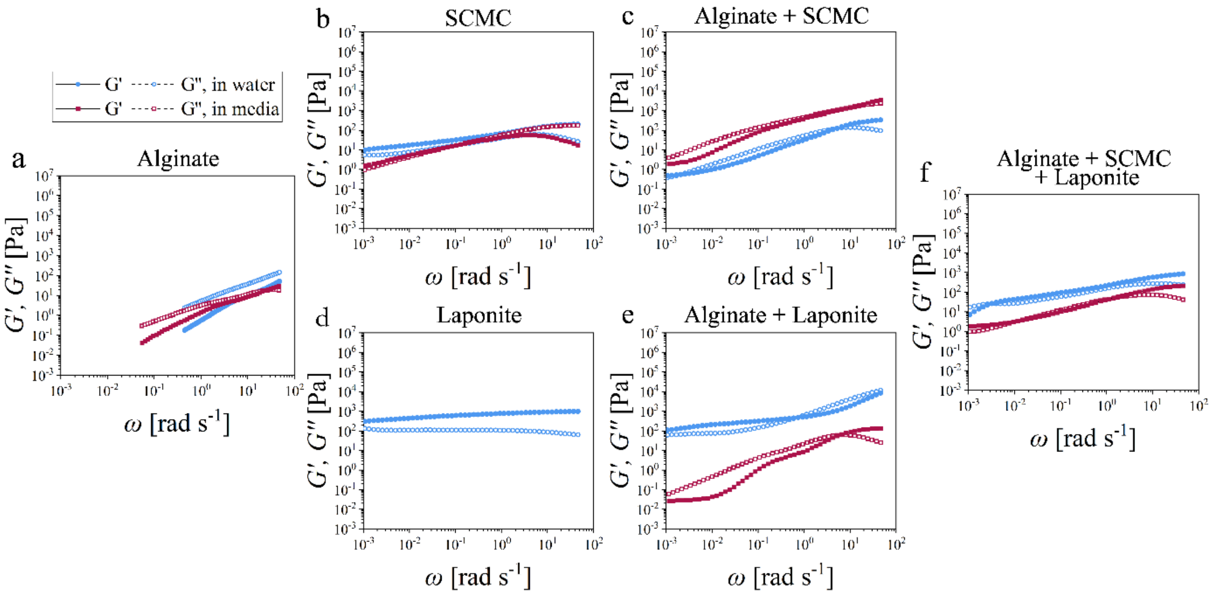

Figure S5. Frequency sweep from relaxation (storage modulus (G') and loss modulus (G'') vs. angular frequency (ω)) at 10 % strain in water and cell culture media for a) pristine alginate 3.5 % (*w/v*), b) pristine sodium carboxymethyl cellulose 3 % (*w/v*), c) blended alginate 3.5 % (*w/v*) with SCMC 3 % (*w/v*), d) pristine laponite-RD 5 % (*w/v*), e) blended alginate 3.5 % (*w/v*) with laponite 5 % (*w/v*), and f) blended alginate 3 % (*w/v*) with SCMC 1.5 % (*w/v*) and laponite-RD 1.5 % (*w/v*) (ratio 2:1:1 (*w/w*)).

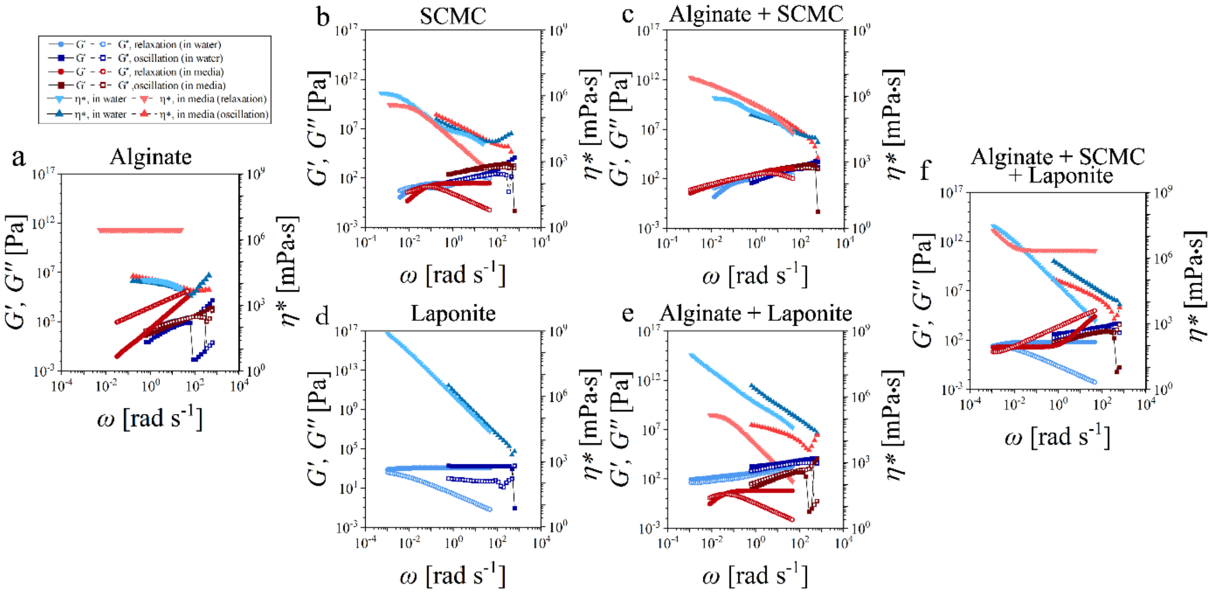

Figure S6. Frequency sweep at 0.1 % shear strain obtained from oscillation and relaxation experiments at 1 % shear strain (storage modulus (G') and loss modulus (G'') vs. angular

frequency (ω)) in water and cell culture media for a) pristine alginate 3.5 % (*w/v*), b) pristine sodium carboxymethyl cellulose 3 % (*w/v*), c) blended alginate 3.5 % (*w/v*) with SCMC 3 % (*w/v*), d) pristine laponite-RD 5 % (*w/v*), e) blended alginate 3.5 % (*w/v*) with laponite 5 % (*w/v*), and f) blended alginate 3 % (*w/v*) with SCMC 1.5 % (*w/v*) and laponite-RD 1.5 % (*w/v*) (ratio 2:1:1 (*w/w*)).

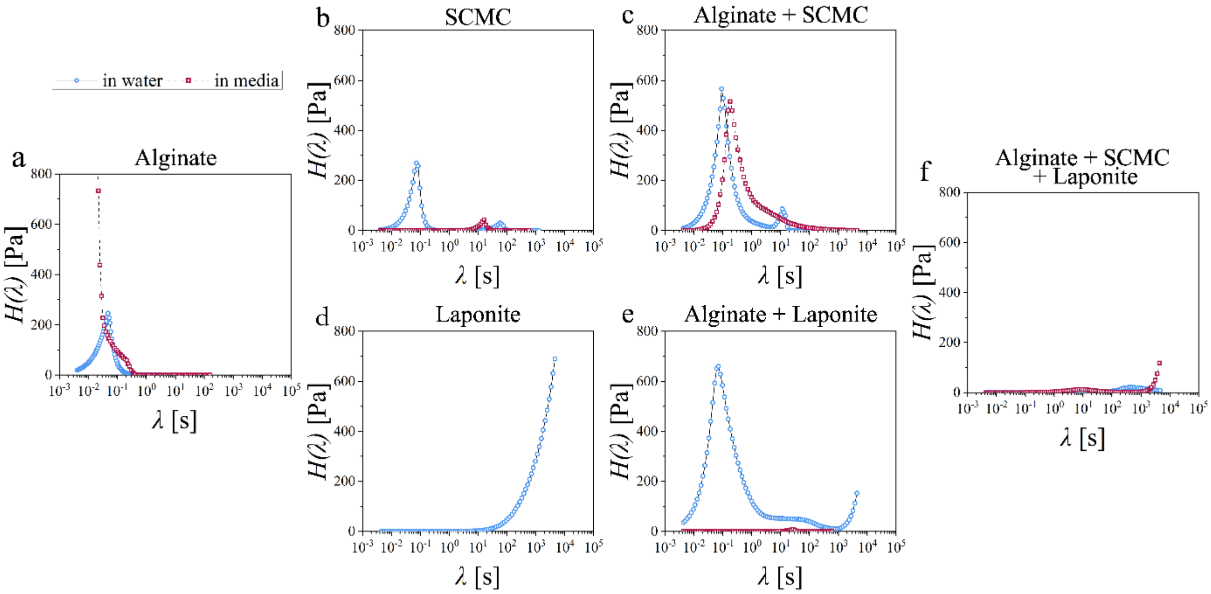

Figure S7. Relaxation time spectrum (relaxation spectrum (H(λ) vs. relaxation time (λ)) at 1 % shear strain in water and cell culture media for a) pristine alginate 3.5 % (*w/v*), b) pristine sodium carboxymethyl cellulose 3 % (*w/v*), c) blended alginate 3.5 % (*w/v*) with SCMC 3 % (*w/v*), d) pristine laponite-RD 5 % (*w/v*), e) blended alginate 3.5 % (*w/v*) with laponite 5 % (*w/v*), and f) blended alginate 3 % (*w/v*) with SCMC 1.5 % (*w/v*) and laponite-RD 1.5 % (*w/v*) (ratio 2:1:1 (*w/w*)).

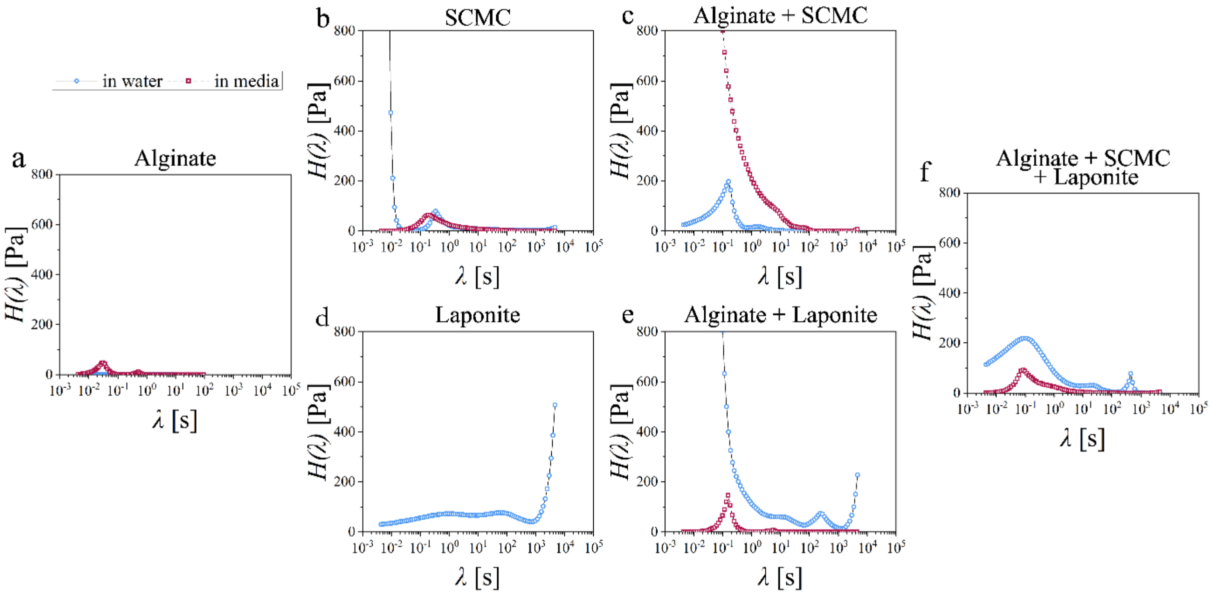

Figure S8. Relaxation time spectrum (relaxation spectrum (H(λ) vs. relaxation time (λ)) at 10 % shear strain in water and cell culture media for a) pristine alginate 3.5 % (*w/v*), b) pristine sodium carboxymethyl cellulose 3 % (*w/v*), c) blended alginate 3.5 % (*w/v*) with SCMC 3 % (*w/v*), d) pristine laponite-RD 5 % (*w/v*), e) blended alginate 3.5 % (*w/v*) with laponite 5 % (*w/v*), and f) blended alginate 3 % (*w/v*) with SCMC 1.5 % (*w/v*) and laponite-RD 1.5 % (*w/v*) (ratio 2:1:1 (*w/w*)).

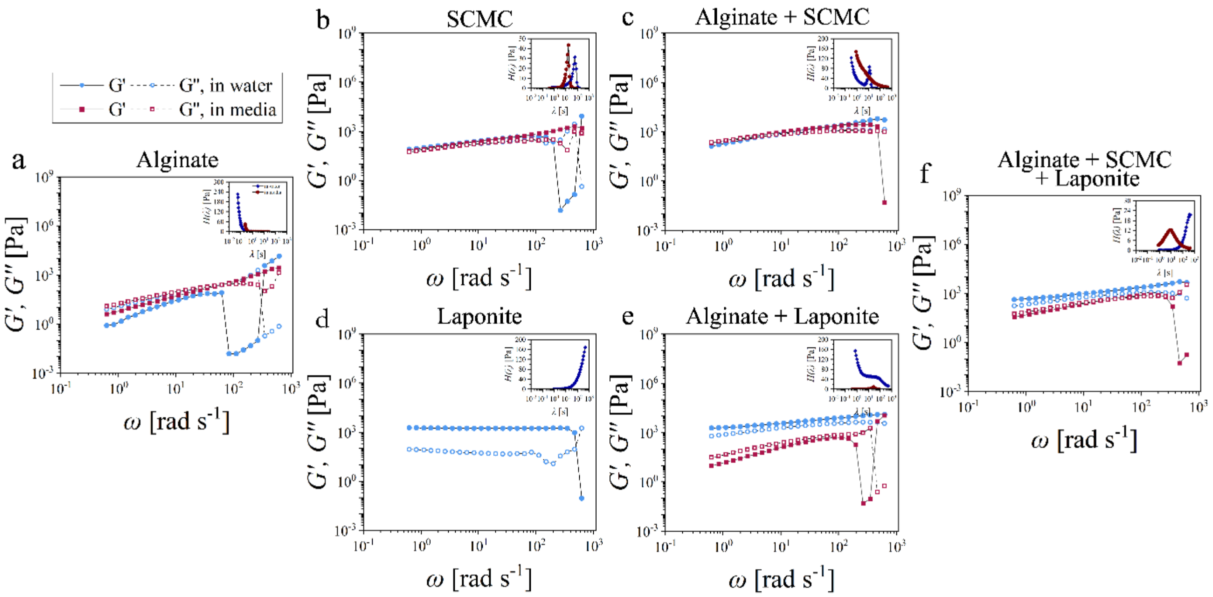

Figure S9. Frequency sweep with relaxation spectrum at 1 % strain in water and cell culture media for a) pristine alginate 3.5 % (*w/v*), b) pristine sodium carboxymethyl cellulose 3 % (*w/v*), c) blended alginate 3.5 % (*w/v*) with SCMC 3 % (*w/v*), d) pristine laponite-RD 5 % (*w/v*), e) blended alginate 3.5 % (*w/v*) with laponite 5 % (*w/v*), and f) blended alginate 3 % (*w/v*) with SCMC 1.5 % (*w/v*) and laponite-RD 1.5 % (*w/v*) (ratio 2:1:1 (*w/w*)).

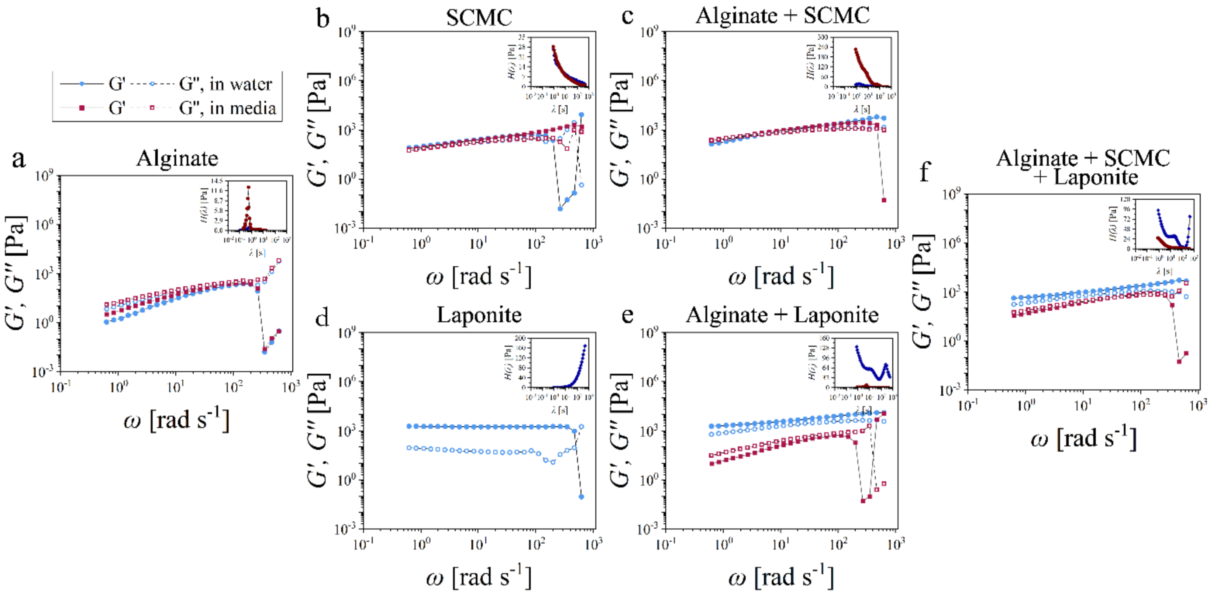

Figure S10. Frequency sweep with relaxation spectrum at 10 % strain in water and cell culture media for a) pristine alginate 3.5 % (*w/v*), b) pristine sodium carboxymethyl cellulose 3 % (*w/v*), c) blended alginate 3.5 % (*w/v*) with SCMC 3 % (*w/v*), d) pristine laponite-RD 5 % (*w/v*), e) blended alginate 3.5 % (*w/v*) with laponite 5 % (*w/v*), and f) blended alginate 3 % (*w/v*) with SCMC 1.5 % (*w/v*) and laponite-RD 1.5 % (*w/v*) (ratio 2:1:1 (*w/w*)).

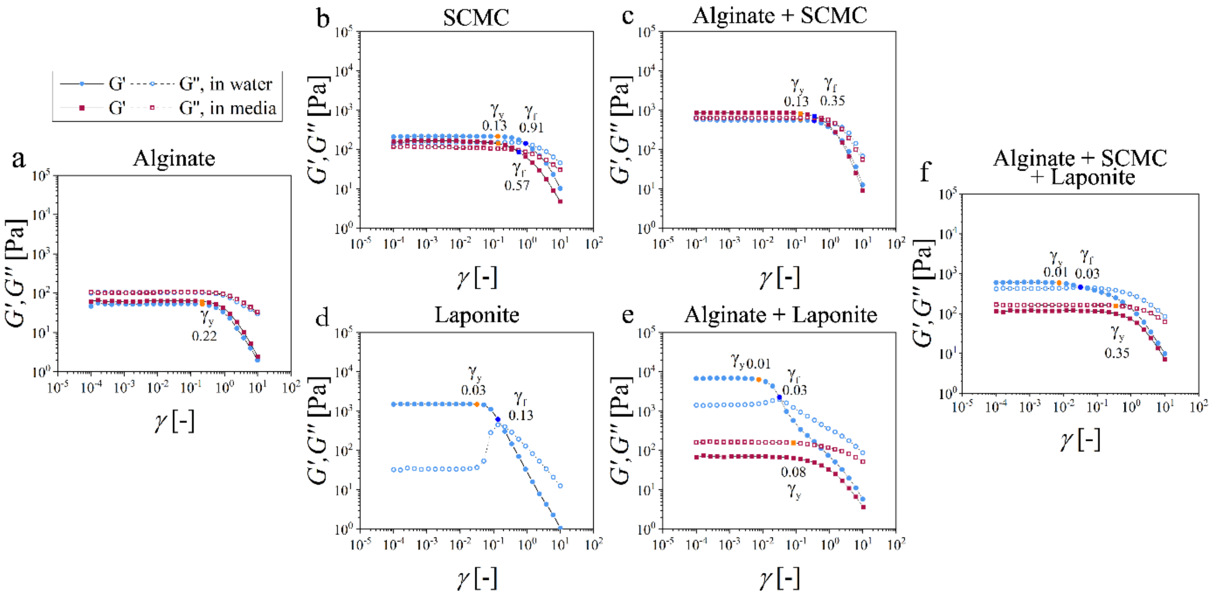

Figure S11. Amplitude sweep (storage modulus (G') and loss modulus (G'') vs. shear strain (γ)) in water and cell culture media for a) pristine alginate 3.5 % (*w/v*), b) pristine sodium carboxymethyl cellulose 3 % (*w/v*), c) blended alginate 3.5 % (*w/v*) with SCMC 3 % (*w/v*), d) pristine laponite-RD 5 % (*w/v*), e) blended alginate 3.5 % (*w/v*) with laponite 5 % (*w/v*), and f) blended alginate 3 % (*w/v*) with SCMC 1.5 % (*w/v*) and laponite-RD 1.5 % (*w/v*) (ratio 2:1:1 (*w/w*)).

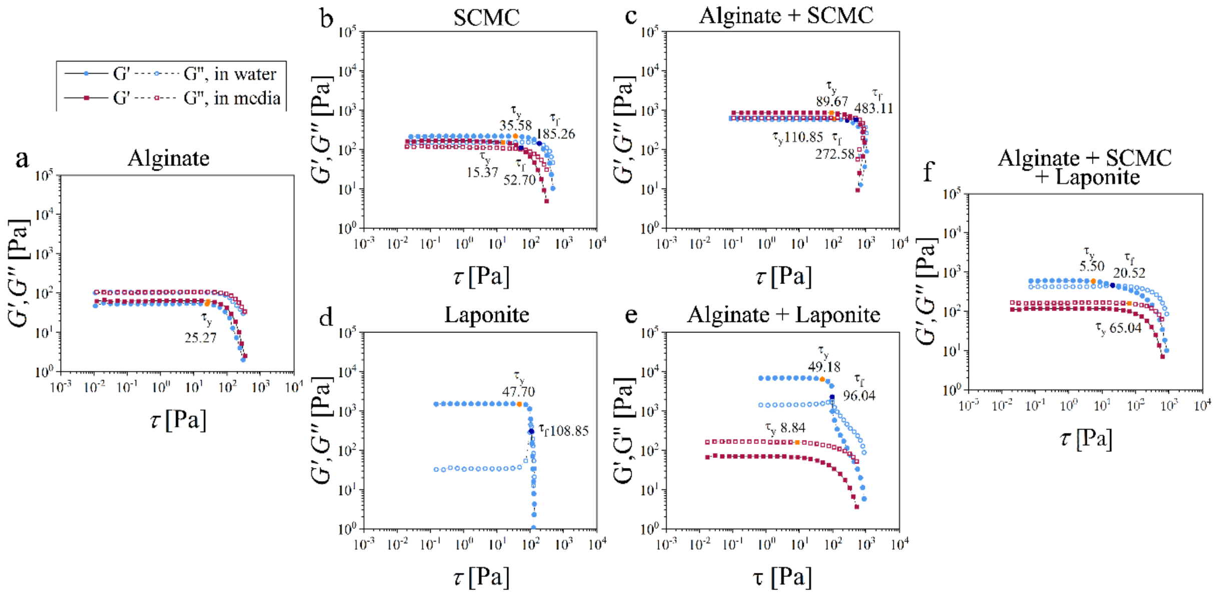

Figure S12. Amplitude sweep at a frequency of 1 Hz, (storage modulus (*G′*) and loss modulus (*G″*) vs. shear stress (τ)) with yield (τ_y) (orange dots) and flow (τ_f) (blue dots) stresses in water and cell culture media for a) pristine alginate 3.5 % (*w/v*), b) pristine sodium carboxymethyl cellulose (SCMC) 3 % (*w/v*), c) blended alginate 3.5 % (*w/v*) with SCMC 3 % (*w/v*), d) pristine laponite-RD 5 % (*w/v*), e) blended alginate 3.5 % (*w/v*) with laponite 5 % (*w/v*), and f) blended alginate 3 % with SCMC 1.5 % (*w/v*) and laponite-RD 1.5 % (*w/v*) (ratio 2:1:1 (*w/w*)).

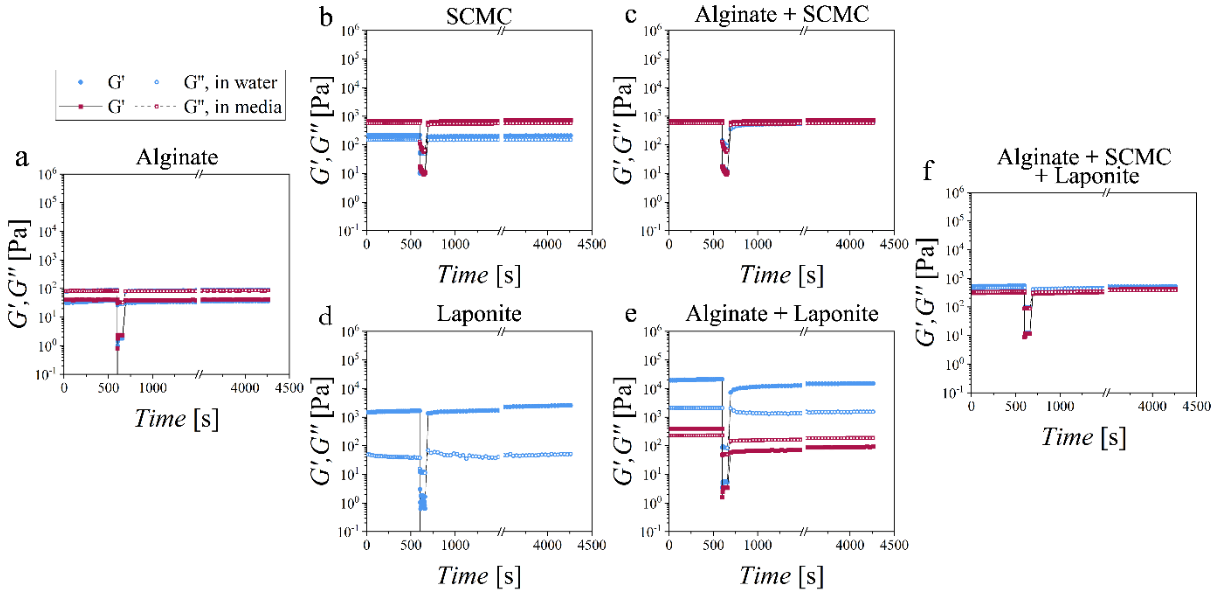

Figure S13. Thixotropy 3 interval test in oscillation mode (storage modulus (*G′*) and loss modulus (*G″*) vs. time (t)) in water and cell culture media for a) pristine alginate 3.5 % (*w/v*), b) pristine sodium carboxymethyl cellulose 3 % (*w/v*), c) blended alginate 3.5 % (*w/v*) with SCMC 3 % (*w/v*), d) pristine laponite-RD 5 % (*w/v*), e) blended alginate 3.5 % (*w/v*) with

laponite 5 % (*w/v*), and f) blended alginate 3 % (*w/v*) with SCMC 1.5 % (*w/v*) and laponite-RD 1.5 % (*w/v*) (ratio 2:1:1 (*w/w*)).

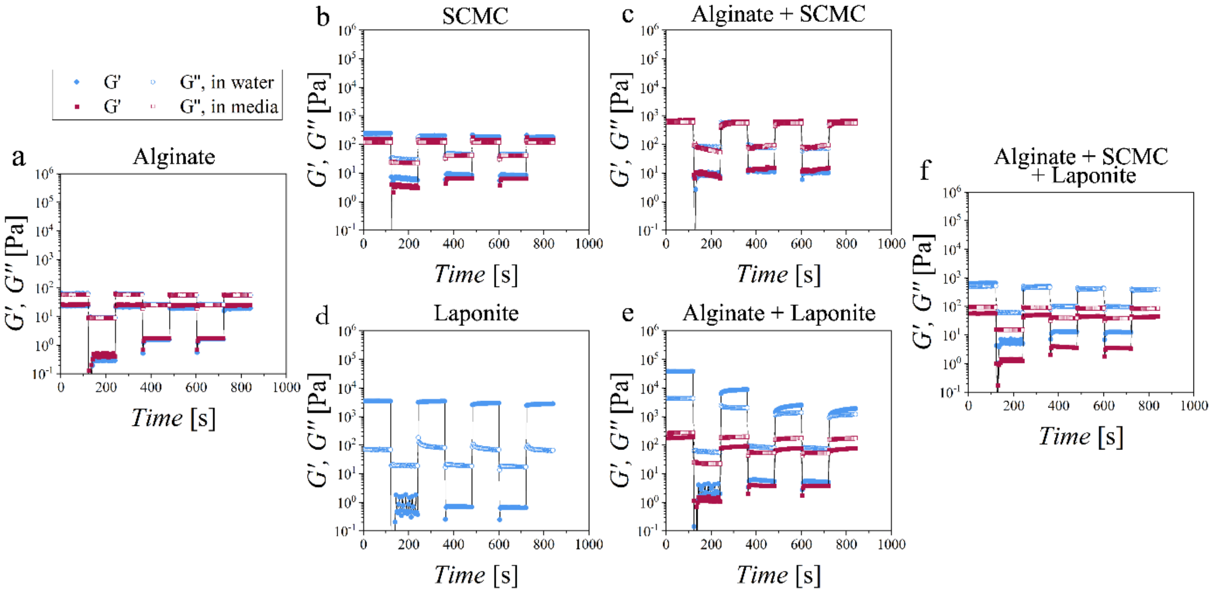

Figure S14. Cyclic thixotropy (3-nterval test) (3ITT) ((storage modulus (G') and loss modulus (G'') vs. time (t)) behavior alternating between shear strains (γ) of 0.1 % and 1000 % in water and cell culture media for a) pristine alginate 3.5 % (*w/v*), b) pristine sodium carboxymethyl cellulose (SCMC) 3 % (*w/v*), c) blended alginate 3.5 % (*w/v*) with SCMC 3 % (*w/v*), d) pristine laponite-RD 5 % (*w/v*), e) blended alginate 3.5 % (*w/v*) with laponite 5 % (*w/v*), and f) blended alginate 3 % with SCMC 1.5 % (*w/v*) and laponite-RD 1.5 % (*w/v*) (ratio 2:1:1 (*w/w*)).

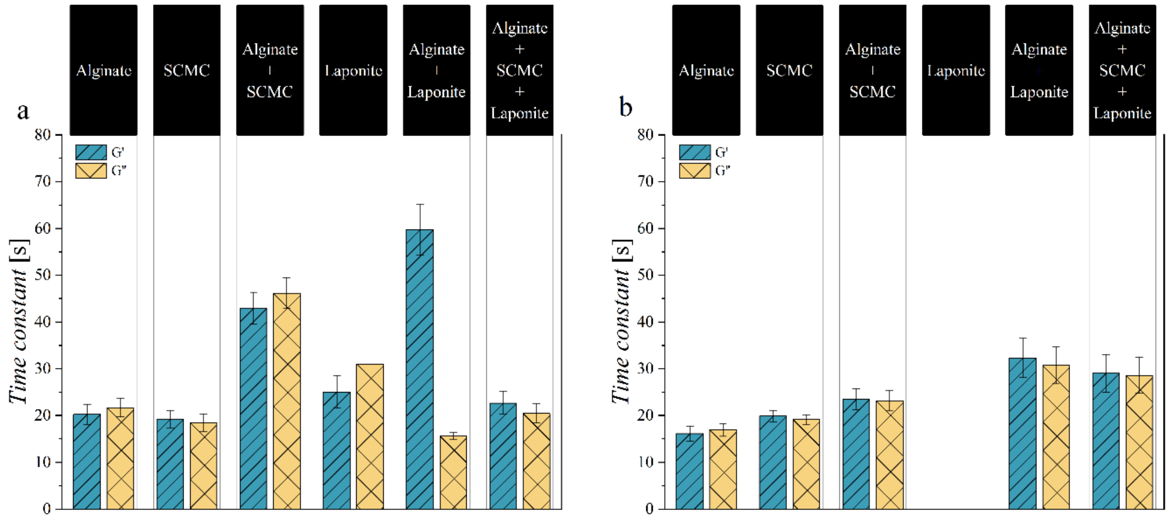

Figure S15. Time constant extracted from exponential decay of the recovery segment of the 3-step thixotropy in a) water and b) cell culture media.

$$y = y_0 + A_1 e^{-x/t_1}$$ **Equation S1**

y_0: offset [Pa]

A_1: Amplitude [-]

t_1: time constant or recovery time [s]

Table S1. One-phase exponential decay fit parameters of the recovery segment of the three-interval test (3ITT) thixotropy test

Solvent type		Water						Media					
Modulus		Storage			Loss			Storage			Loss		
Hydrogel type	Concentration	t	A	Y_0	t	A	Y_0	t	A	Y_0	t	A	Y_0
Alg	3.5 %	20	$-4x10^{15}$	34	22	$-8x10^{14}$	34	16	$-2x10^{19}$	40	17	$-4x10^{18}$	81
SCMC	3 %	19	$-1x10^{17}$	201	18	$-3x10^{17}$	151	20	$-2x10^{17}$	680	19	$-4x10^{17}$	548
Lap	5 %	25	$-4x10^{14}$	1632	31	$-6x10^{7}$	46	-	-	-	-	-	-
Alg + SCMC + Lap	3 + 1.5 + 1.5 %	23	$-2x10^{15}$	441	20	$-3x10^{16}$	432	29	$-2x10^{12}$	319	29	$-2x10^{12}$	338
Alg + SCMC	3.5 + 3 %	43	$-2x10^{9}$	528	46	$-7x10^{8}$	541	24	$-9x10^{14}$	681	23	$-1x10^{15}$	549
Alg + Lap	3.5 + 5 %	60	$-7x10^{8}$	12067	16	$-3x10^{21}$	1423	32	$-4x10^{10}$	69	31	$-2x10^{11}$	158

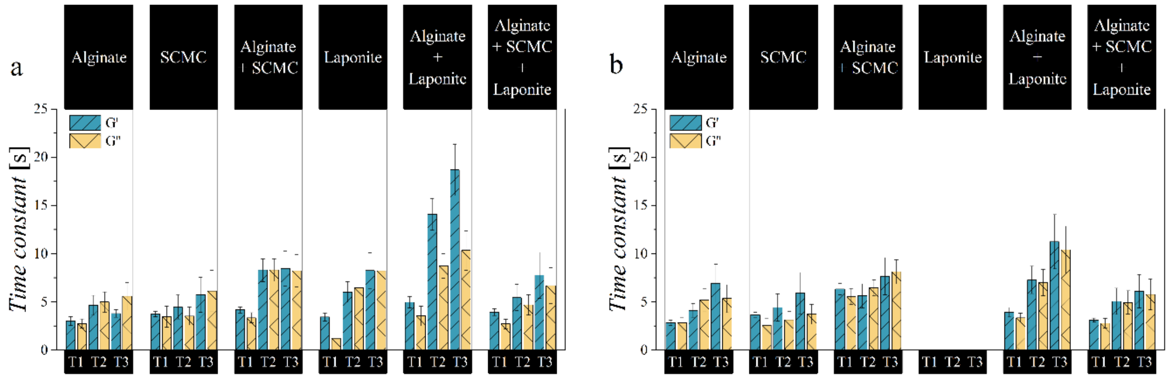

Figure S16. Time constant obtained from one-phase exponential decay fit of the recovery segment of cyclic thixotropy in a) water and b) cell culture media within the three intervals (T1, T2, and T3).

Table S2. One-phase exponential decay fit parameters of the recovery segment for the first cycle of the cyclic thixotropy 3ITT

Solvent type		Water						Media					
Modulus		Storage			Loss			Storage			Loss		
Hydrogel type	Concentration	t	A	Y_0	t	A	Y_0	t	A	Y_0	t	A	Y_0
Alg	3.5 %	3	$-7x10^{35}$	22	3	$-2x10^{39}$	62	3	-2E+38	26	3	$-3x10^{38}$	59
SCMC	3 %	4	$-2x10^{30}$	203	3	$-1x10^{32}$	153	4	-6E+30	147	3	$-5x10^{42}$	120
Alg + SCMC	3.5 + 3 %	4	$-5x10^{27}$	587	3	$-5x10^{33}$	605	6	-2E+19	623	6	$-2x10^{21}$	541
Lap	5 %	3	$-6x10^{33}$	3415	1	$-2x10^{5}$	84	-	-	-	-	-	-
Alg + Lap	3.5 + 5 %	5	$-8x10^{24}$	8413	4	$-4x10^{32}$	2110	4	-3E+28	89	3	$-2x10^{33}$	191

Alg + SCMC + Lap	3 + 1.5 + 1.5 %	4	$-1x10^{29}$	494	3	$-9x10^{40}$	471	3	-1E+35	50	3	$-1x10^{39}$	92

Table S3. One-phase exponential decay fit parameters of the recovery segment for the second cycle of the cyclic thixotropy 3 ITT

	**Solvent type**	**Water**						**Media**					
	**Modulus**	**Storage**			**Loss**			**Storage**			**Loss**		
**Hydrogel type**	**Concentration**	**t**	**A**	**Y_0**	**t**	**A**	**Y_0**	**t**	**A**	**Y_0**	**t**	**A**	**Y_0**
Alg	3.5 %	5	$-8x10^{45}$	20	5	$-1x10^{43}$	56	4	$-6x10^{51}$	26	5	$-3x10^{41}$	57
SCMC	3 %	4	$-7x10^{48}$	189	4	$-8x10^{60}$	144	4	$-1x10^{49}$	147	3	$-1x10^{68}$	118
Alg + SCMC	3.5 + 3 %	8	$-5x10^{39}$	658	8	$-8x10^{34}$	558	6	$-5x10^{39}$	658	6	$-8x10^{34}$	558
Lap	5 %	6	$-10x10^{37}$	2951	6	$-5x10^{33}$	77	-	-	-	-	-	-
Alg + Lap	3.5 + 5 %	14	$-1x10^{18}$	2342	9	$-6x10^{26}$	1340	7	$-3x10^{30}$	76	7	$-4x10^{31}$	172
Alg + SCMC + Lap	3 + 1.5 + 1.5 %	5	$-5x10^{40}$	422	5	$-8x10^{46}$	420	5	$-4x10^{42}$	45	5	$-5x10^{43}$	86

Table S4. One-phase exponential decay fit parameters of the recovery segment for the third cycle of the cyclic thixotropy 3 ITT

	**Solvent type**	**Water**						**Media**					
	**Modulus**	**Storage**			**Loss**			**Storage**			**Loss**		
**Hydrogel type**	**Concentration**	**t**	**A**	**Y_0**	**t**	**A**	**Y_0**	**t**	**A**	**Y_0**	**t**	**A**	**Y_0**
Alg	3.5 %	4	$-2x10^{83}$	19	6	$-2x10^{57}$	56	7	$-2x10^{46}$	25	5	$-7x10^{59}$	57
SCMC	3 %	6	$-4x10^{56}$	185	6	$-7x10^{52}$	141	6	$-8x10^{54}$	147	4	$-5x10^{85}$	117
Alg + SCMC	3.5 + 3 %	8	$-4x10^{39}$	609	8	$-3x10^{40}$	588	8	$-3x10^{43}$	676	8	$-1x10^{41}$	558
Lap	5 %	8	$-2x10^{41}$	2778	8	$-2x10^{39}$	71	-	-	-	-	-	-
Alg + Lap	3.5 + 5 %	19	$-8x10^{19}$	1808	10	$-1x10^{33}$	1162	11	$-3x10^{29}$	75	10	$-9x10^{31}$	170
Alg + SCMC + Lap	3 + 1.5 + 1.5 %	8	$-4x10^{42}$	390	7	$-1x10^{49}$	397	6	$-5x10^{52}$	44	6	$-5x10^{55}$	84

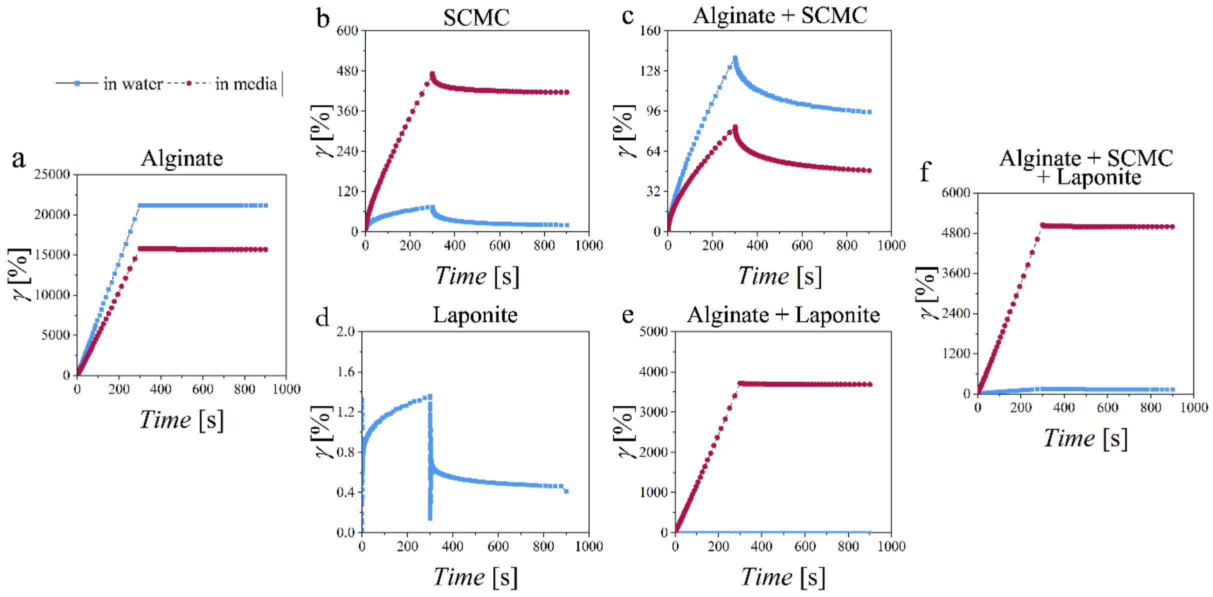

Figure S17. Creep-Recovery (shear strain (*γ*) vs. time (t)) at a stress of 10 Pa in water and cell culture media for a) pristine alginate 3.5 % (*w/v*), b) pristine sodium carboxymethyl cellulose 3 % (*w/v*), c) blended alginate 3.5 % (*w/v*) with SCMC 3 % (*w/v*), d) pristine laponite-RD 5 % (*w/v*), e) blended alginate 3.5 % (*w/v*) with laponite 5 % (*w/v*), and f) blended alginate 3 % (*w/v*) with SCMC1.5 % (*w/v*) and laponite-RD 1.5 % (*w/v*) (ratio 2:1:1 (*w/w*)).

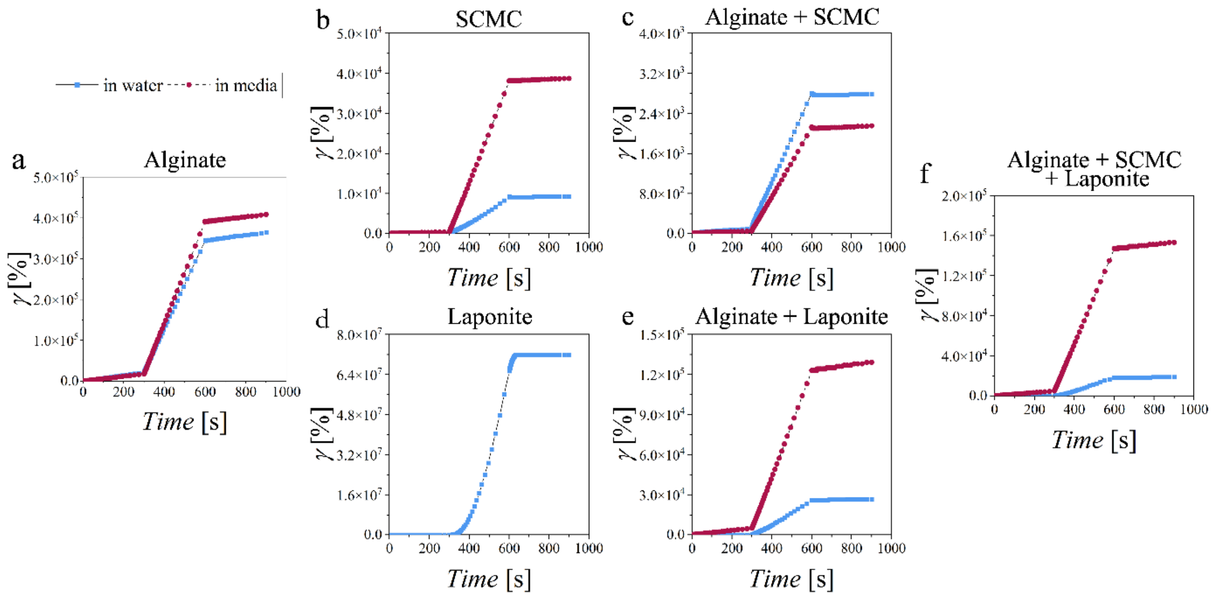

Figure S18. Creep – recovery test (3-interval test) (shear strain (*γ*) vs. time (t)) in water and cell culture media for a) pristine alginate 3.5 % (*w/v*), b) pristine sodium carboxymethyl cellulose 3 % (*w/v*), c) blended alginate 3.5 % (*w/v*) with SCMC 3 % (*w/v*), d) pristine laponite-RD 5 % (*w/v*), e) blended alginate 3.5 % (*w/v*) with laponite 5 % (*w/v*), and f) blended alginate 3 % (*w/v*) with SCMC 1.5 % (*w/v*) and laponite-RD 1.5 % (*w/v*) (ratio 2:1:1 (*w/w*)).

$$\eta_0 = \frac{\tau_0}{\Delta\gamma/\Delta t} \times 100$$ **Equation S2**

η_0 : apparent viscosity [Pa s]

τ_0 : applied stress [Pa]

$\Delta\gamma$: deformation [-]

Δt : time [s]

Rotational experiments:

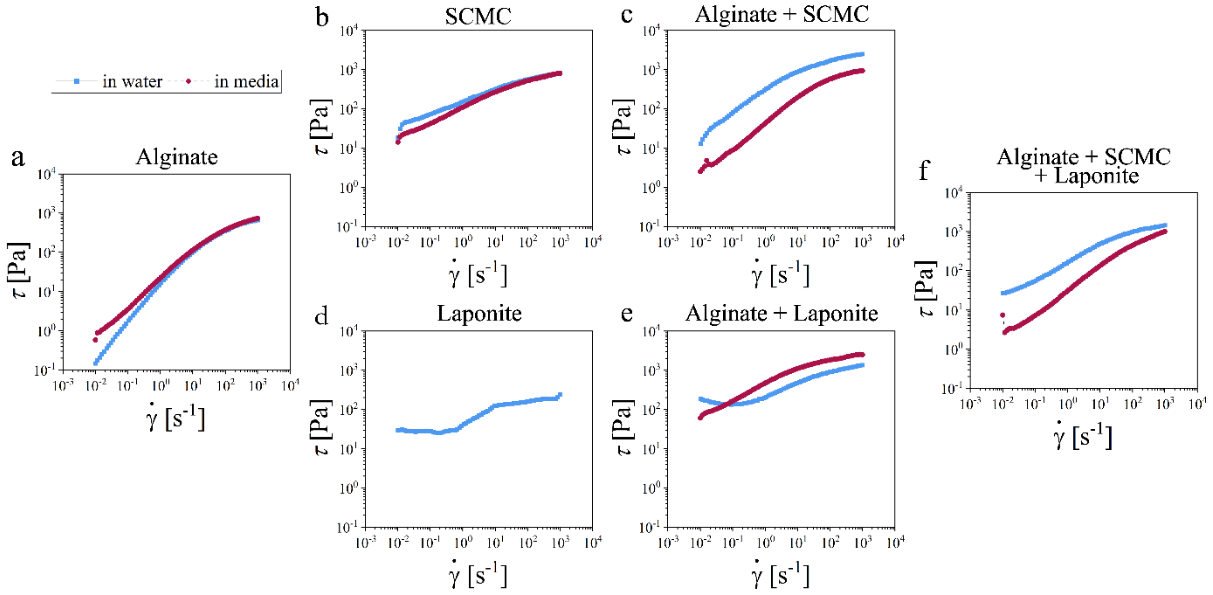

Figure S19. Flow curve or steady-state shear rate ramp (shear stress (τ) vs. shear rate ($\dot{\gamma}$)) in water and cell culture media for a) pristine alginate 3.5 % (*w/v*), b) pristine sodium carboxymethyl cellulose 3 % (*w/v*), c) blended alginate 3.5 % (*w/v*) with SCMC 3 % (*w/v*), d) pristine laponite-RD 5 % (*w/v*), e) blended alginate 3.5 % (*w/v*) with laponite 5 % (*w/v*), and f) blended alginate 3 % (*w/v*) with SCMC 1.5 % (*w/v*) and laponite-RD 1.5 % (*w/v*) (ratio 2:1:1 (*w/w*)).

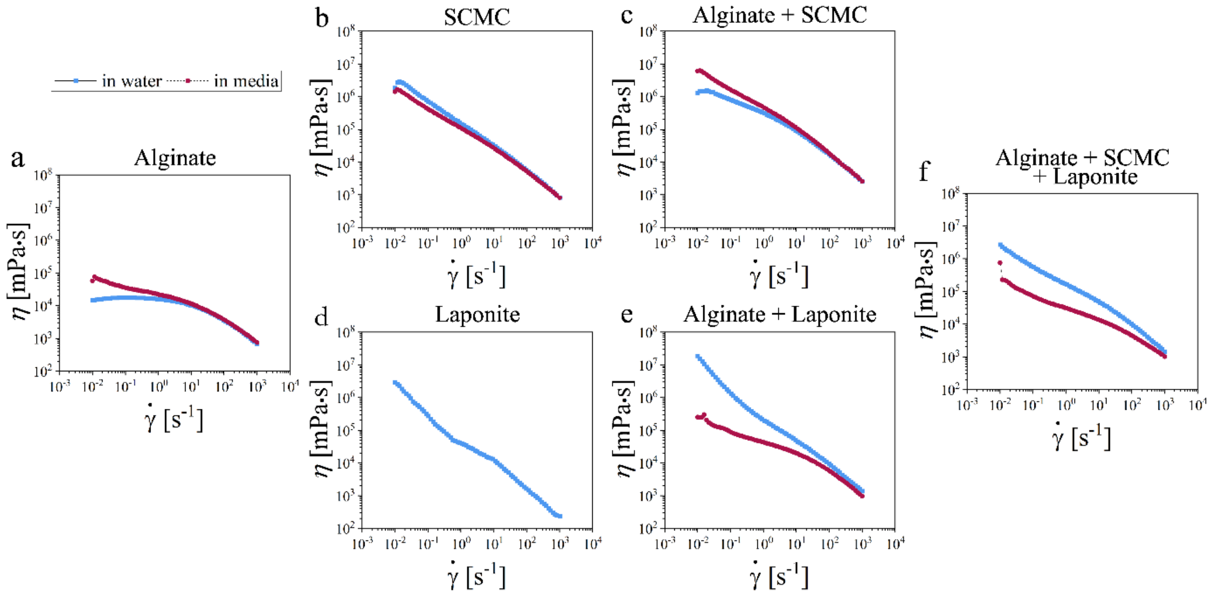

Figure S20. Flow curve or steady-state shear rate ramp (viscosity (η) vs. shear rate ($\dot{\gamma}$)) in water and cell culture media for a) pristine alginate 3.5 % (*w/v*), b) pristine sodium carboxymethyl cellulose 3 % (*w/v*), c) blended alginate 3.5 % (*w/v*) with SCMC 3 % (*w/v*), d) pristine laponite-

RD 5 % (*w/v*), e) blended alginate 3.5 % (*w/v*) with laponite 5 % (*w/v*), and f) blended alginate 3 % (*w/v*) with SCMC 1.5 % (*w/v*) and laponite-RD 1.5 % (*w/v*) (ratio 2:1:1 (*w/w*)).

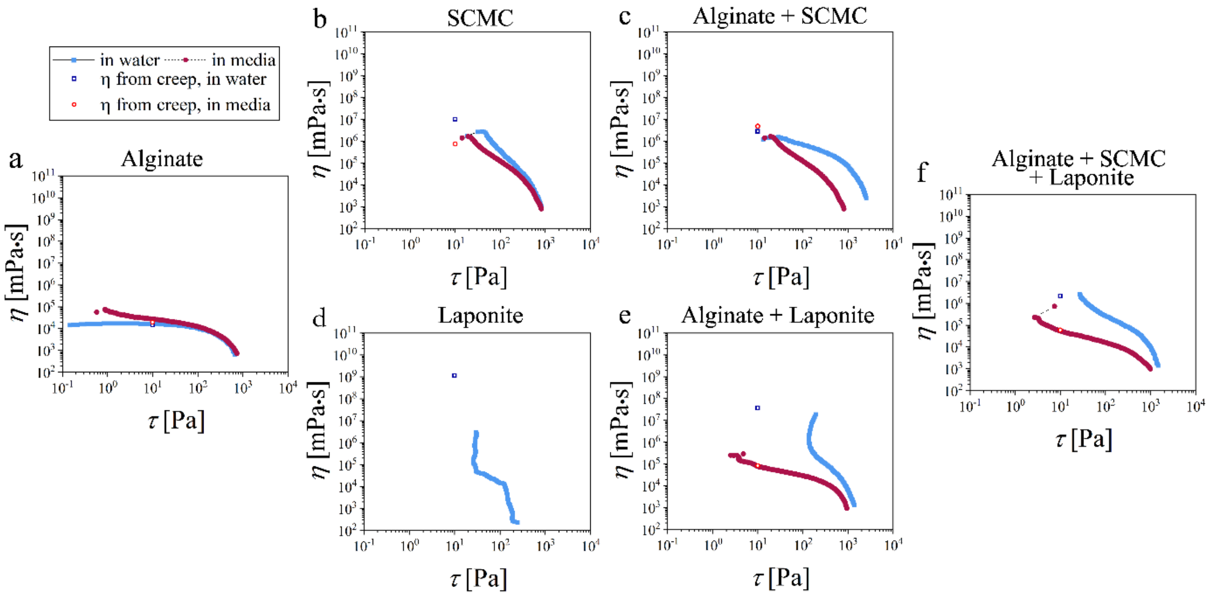

Figure S21. Flow curve or steady-state shear rate ramp (viscosity (η) vs. shear stress (τ)) in water and cell culture media for a) pristine alginate 3.5 % (*w/v*), b) pristine sodium carboxymethyl cellulose 3 % (*w/v*), c) blended alginate 3.5 % (*w/v*) with SCMC 3 % (*w/v*), d) pristine laponite-RD 5 % (*w/v*), e) blended alginate 3.5 % (*w/v*) with laponite 5 % (*w/v*), and f) blended alginate 3 % (*w/v*) with SCMC 1.5 % (*w/v*) and laponite-RD 1.5 % (*w/v*) (ratio 2:1:1 (*w/w*)).

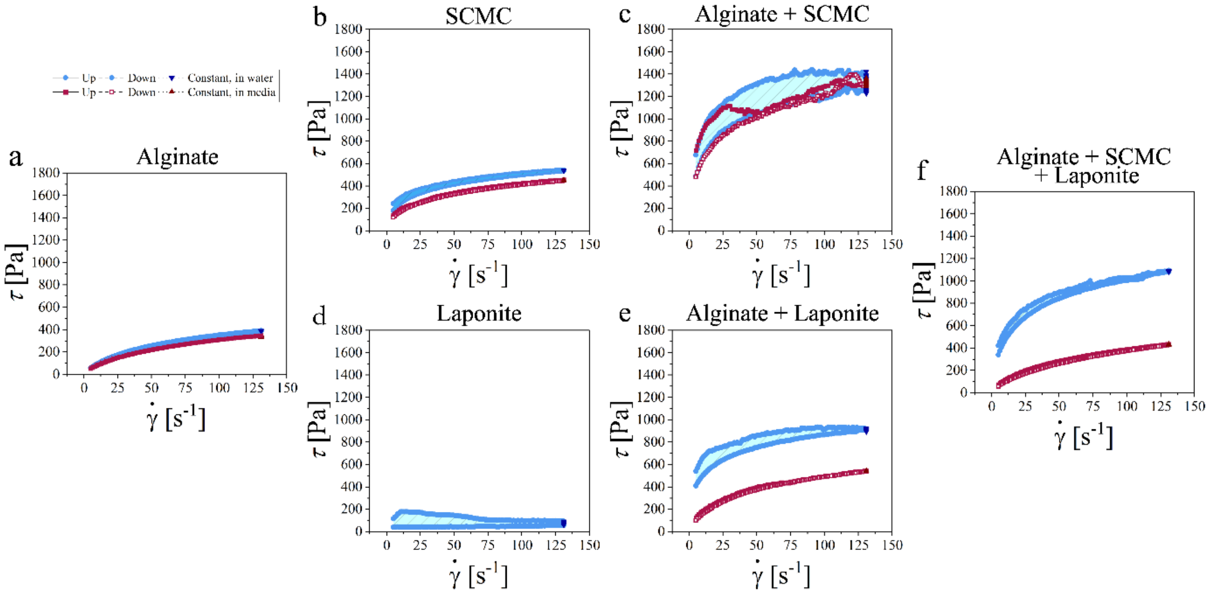

Figure S22. Hysteresis loop or shear rate ramp up-constant-down (shear stress (τ) vs. shear rate ($\dot{\gamma}$)) in water and cell culture media for a) pristine alginate 3.5 % (*w/v*), b) pristine sodium carboxymethyl cellulose 3 % (*w/v*), c) blended alginate 3.5 % (*w/v*) with SCMC 3 % (*w/v*), d)

pristine laponite-RD 5 % (*w/v*), e) blended alginate 3.5 % (*w/v*) with laponite 5 % (*w/v*), and f) blended alginate 3 % (*w/v*) with SCMC 1.5 % (*w/v*) and laponite-RD 1.5 % (*w/v*) (ratio 2:1:1 (*w/w*)).

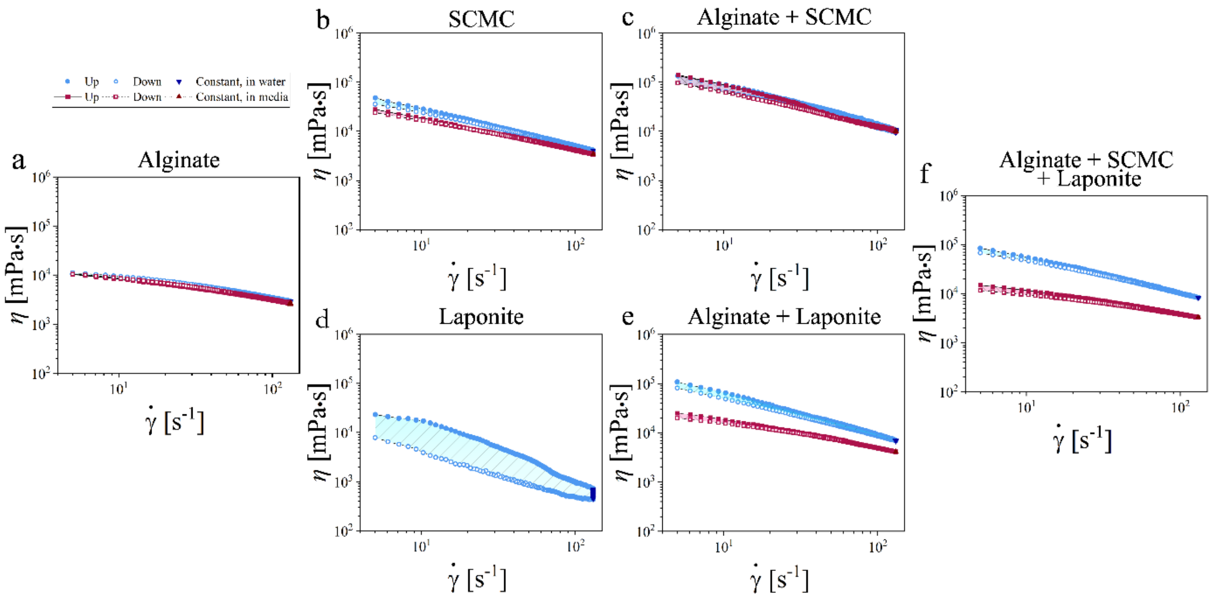

Figure S23. Hysteresis loop or shear rate ramp up-constant-down (viscosity (η) vs. shear rate ($\dot{\gamma}$)) in water and cell culture media for a) pristine alginate 3.5 % (*w/v*), b) pristine sodium carboxymethyl cellulose 3 % (*w/v*), c) blended alginate 3.5 % (*w/v*) with SCMC 3 % (*w/v*), d) pristine laponite-RD 5 % (*w/v*), e) blended alginate 3.5 % (*w/v*) with laponite 5 % (*w/v*), and f) blended alginate 3 % (*w/v*) with SCMC 1.5 % (*w/v*) and laponite-RD 1.5 % (*w/v*) (ratio 2:1:1 (*w/w*)).

Cell culture experiments:

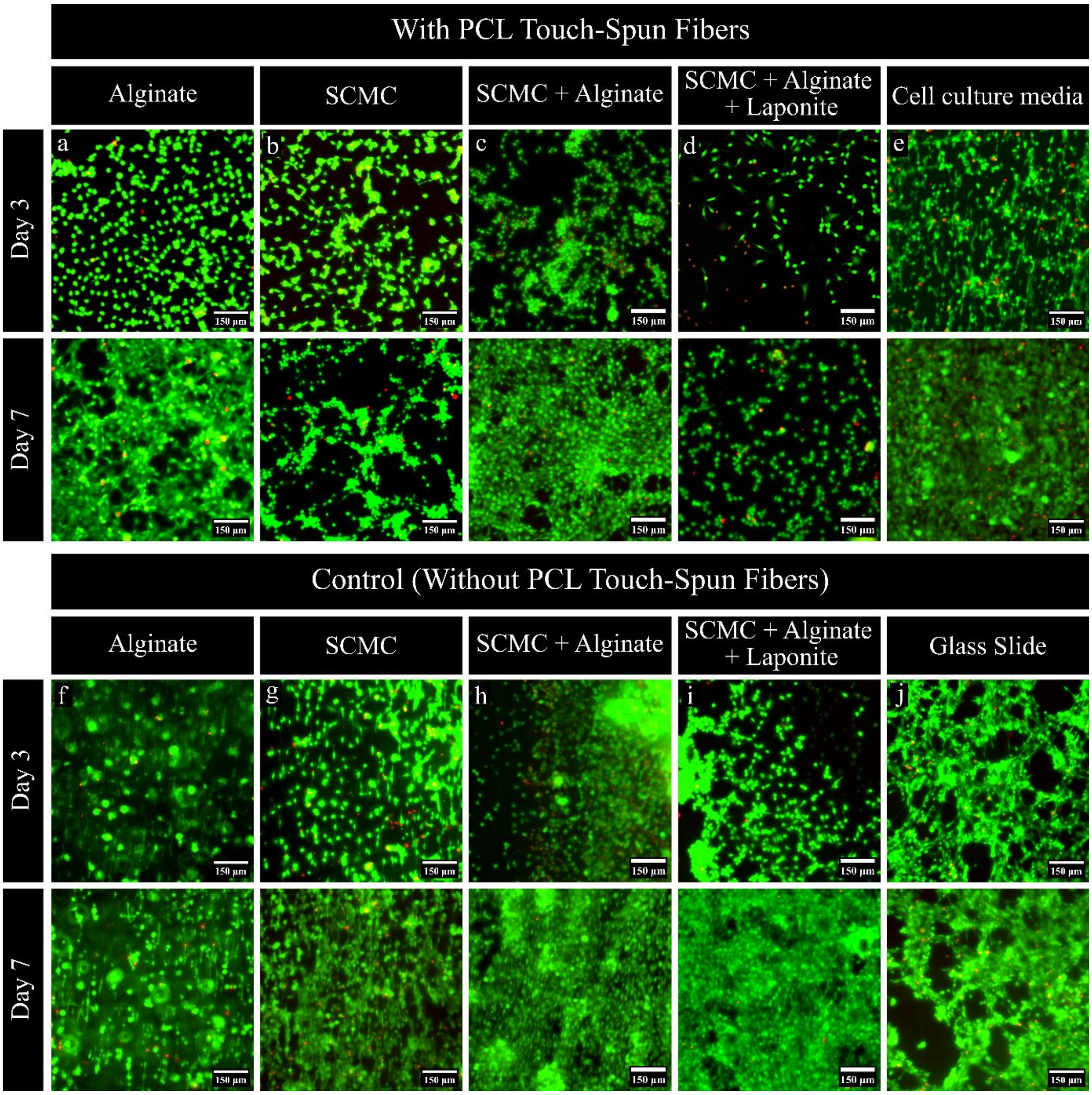

Figure S24. Cell viability assay using Calcein AM and ethidium homodimer-1, where live cells are shown in green and dead cells are shown in red, a-e) with PCL touch-spun fibers and f-j) controls (with PCL touch-spun fibers, scale bar 150 µm).

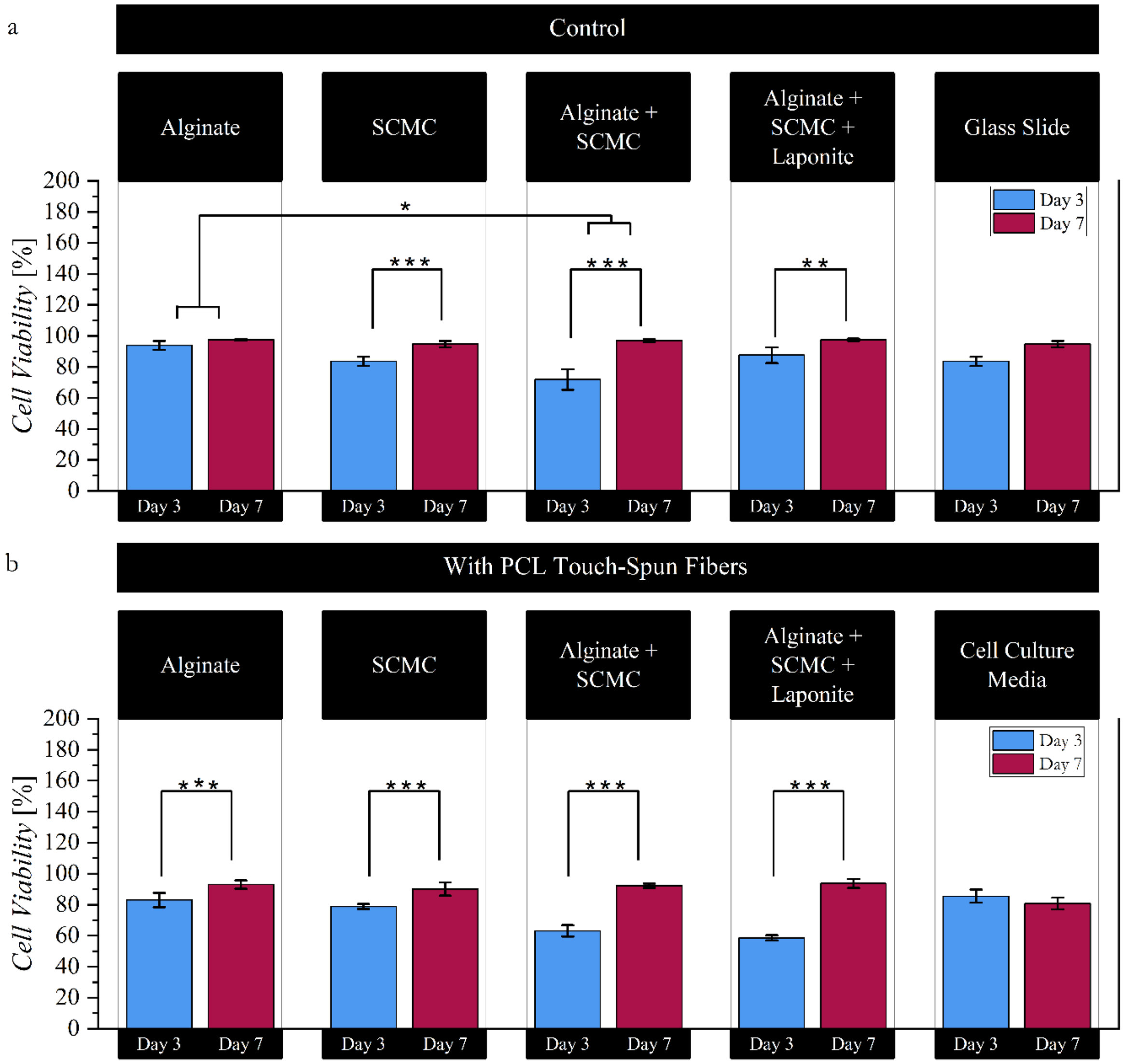

Figure S25. Fibroblasts viability, where a) controls (cells suspended in hydrogels without fibers and cells directly seeded on glass slide) and b) the multilayered 3D bioprinted bioink - PCL touch-spun fibers constructs and PCL-touch spun fibers alone (cells suspended in cell culture media) (mean ± SD; * $p \leq 0.05$, ** $p \leq 0.01$, *** $p \leq 0.001$; $n = 5$).

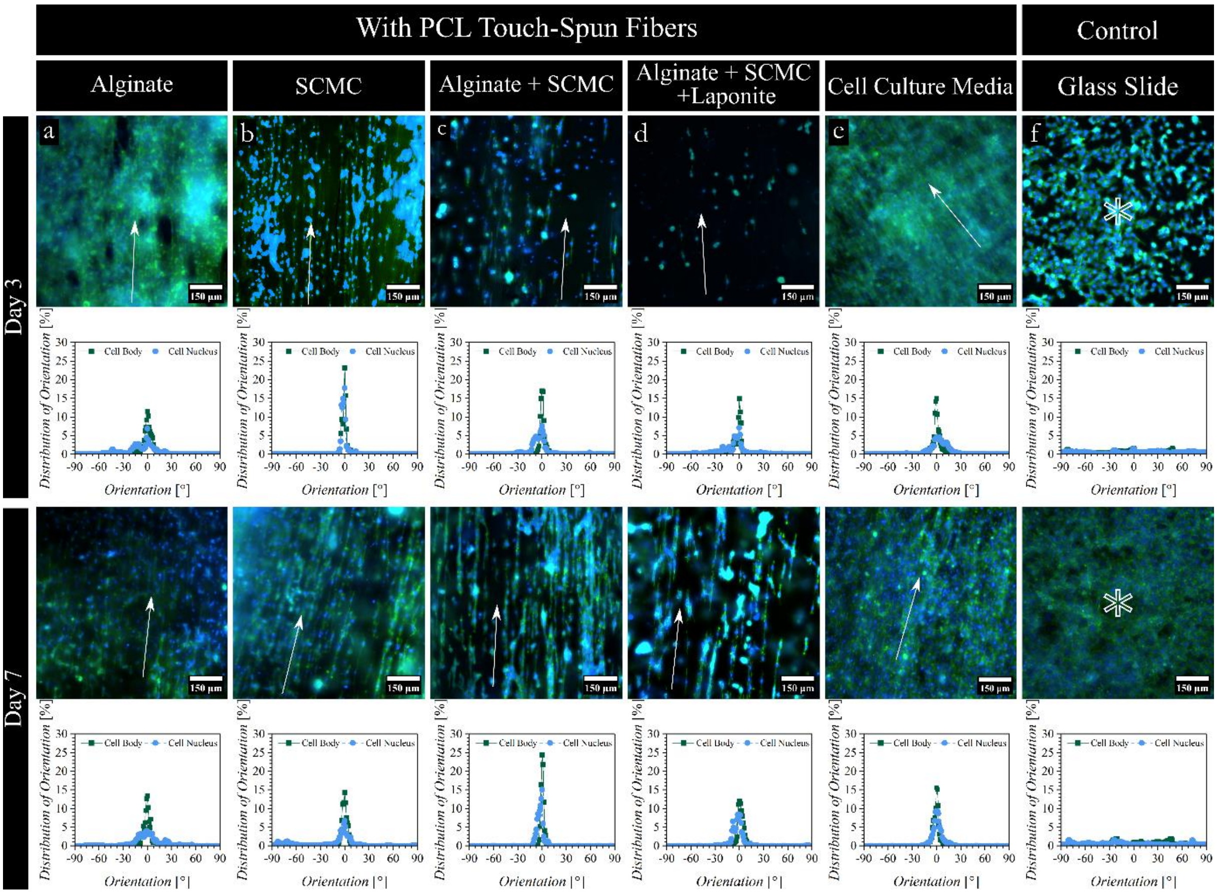

Figure S26. Fibroblast cells orientation at a-f) day 3 and 7 of incubation, green is the cell body (actin filaments), blue is the cell nucleus, and arrows indicate the main direction of fibers (0°). Fluorescence microscopy images were taken at 20x magnification, scale bar 150 µm.

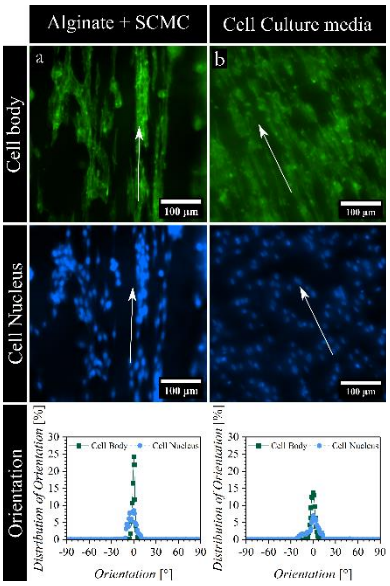

Figure S27. Fibroblast cells orientation at day 7 of incubation on multilayered 3D bioprinted bioink - PCL touch-spun fibers construct, green is cell body (actin filaments), blue is cell nucleus, and arrows indicate the main direction of fibers (0°), a) alginate with SCMC as bioink

material and b) without bioink (PCL fibers alone with cells suspended in cell culture media). Fluorescence microscopy images were taken at 40x magnification, scale bar 100 μm.

Extrusion videos:

S1 Alginate water

S2 Alginate media

S3 SCMC water

S4 SCMC media

S5 Alginate + SCMC water

S6 Alginate + SCMC media

S7 Laponite water

S8 Alginate + Laponite water

S9 Alginate + Laponite media

S10 Alginate + SCMC + Laponite water

S11 Alginate + SCMC + Laponite media

Time-Lapse videos:

S12 Alginate

S13 SCMC

S14 Alginate + SCMC

S15 Alginate + SCMC + Laponite

S16 PCL
